# Defining Coordinated Care for People with Rare Conditions: A Scoping Review

**DOI:** 10.5334/ijic.5464

**Published:** 2020-06-25

**Authors:** Holly Walton, Emma Hudson, Amy Simpson, Angus I. G. Ramsay, Joe Kai, Stephen Morris, Alastair G. Sutcliffe, Naomi J. Fulop

**Affiliations:** 1 Department of Applied Health Research, University College London, London, UK; 2 Department of Public Health and Primary Care, University of Cambridge, Institute of Public Health, Forvie Site, Robinson Way, Cambridge, UK; 3 Genetic Alliance UK, London, UK; 4 School of Medicine, University of Nottingham, Division of Primary Care, Nottingham, UK; 5 UCL and Great Ormond Street Institute of Child Health, London, UK

**Keywords:** coordinated care, chronic conditions, rare conditions, definition, components, scoping review

## Abstract

**Introduction:**

To coordinate care effectively for rare conditions, we need to understand what coordinated care means. This review aimed to define coordinated care and identify components of coordinated care within the context of rare diseases; by drawing on evidence from chronic conditions.

**Methods:**

A systematic scoping review. We included reviews that reported or defined and outlined components of coordinated care for chronic or rare conditions. Thematic analysis was used to develop a definition and identify components or care coordination. Stakeholder consultations (three focus groups with patients, carers and healthcare professionals with experience of rare conditions) were held to further explore the relevance of review findings for rare conditions.

**Results:**

We included 154 reviews (n = 139 specific to common chronic conditions, n = 3 specific to rare conditions, n = 12 both common/rare conditions). A definition of coordination was developed. Components were identified and categorised by those that: may need to be coordinated, inform how to coordinate care, have multiple roles, or that contextualise coordination.

**Conclusions:**

Coordinated care is multi-faceted and has both generic and context-specific components. Findings outline many ways in which care may be coordinated for both rare and common chronic conditions. Findings can help to develop and eventually test different ways of coordinating care for people with rare and common chronic conditions.

## Introduction

Living with a rare or common chronic condition can have a large impact on a person’s life, including their emotional and psychological health and social and financial situation [1,2,3,4]. Accessing treatment for rare or common chronic conditions may be difficult if there are requirements to attend multiple health appointments, with different specialists, on different days or locations [5, Berenson, 2007; as cited in 6,7,8]. The impact of living with these conditions and experiences accessing treatment are often exacerbated by disjointed care, for example, needing to repeat information to different providers [5,9]. To ease treatment burden and difficulties associated with disjointed care, and make it easier for people to access treatment, it has been argued that there are benefits to coordinating care [5,7,10].

Collectively, rare conditions affect a significant proportion of the population, both in the UK [7] and worldwide [11]. Rare conditions (including undiagnosed conditions) are often genetic disorders, can include both intellectual and physical health symptoms, and largely affect children [3,12]. The European definition proposes that rare conditions are conditions which have been diagnosed in fewer than five in 10,000 people in the general population [3,7]. Many, if not most, rare conditions are chronic, life-long conditions. ‘Chronic disease’ is an umbrella term used to refer to a range of long-term conditions (e.g. cardiovascular disease, cancers, chronic respiratory diseases and diabetes) [13,14]. Chronic conditions may include both common and rare conditions.

In order to understand how care can be coordinated for patients with rare conditions, a clear definition of care coordination is needed in the context of rare conditions. A definition could further our understanding of care coordination for rare diseases and could also help to identify situations where services are not coordinated, and as such may require improvement. A systematic review of reviews conducted by McDonald et al. [15] defined care coordination as “the deliberate organisation of patient care activities between two or more participants (including the patient) involved in a patient’s care to facilitate the appropriate delivery of health care services” [15, p.^18^]. However, many terms and definitions have been used to refer to care coordination, including ‘care management’, ‘integrated care’, and ‘collaboration’ [15].

Identifying key coordination components can help support development of care coordination programs and evaluation of whether these components are delivered in practice as planned [16]. This may contribute towards a better understanding of whether and how these programs are effective. The clear identification of components may also help to standardise the delivery of such programs (where appropriate) to avoid variation in effectiveness due to differing implementation across settings [17]. It may also help to have an understanding of which components are applicable across chronic conditions and which are most relevant for rare conditions. The detailed identification of components could inform evaluations of the potential costs of implementing care coordination for rare conditions within the healthcare system. Earlier reviews have identified and grouped components for long-term conditions. For example: McDonald et al. [15] grouped components into: essential care tasks, associated coordination activities and common features of interventions to support coordination. Whereas, Davies et al. [18] identified nine coordination strategies: communication between service providers, use of systems to support care coordination, coordinating clinical activities, support for providers, support for patients, relationships between service providers, joint planning, funding/management, agreement between organisations and organisation of healthcare systems. One challenge associated with identifying coordination components is that it is difficult to distinguish between processes of care and coordination components, as care coordination may be interlinked with quality, delivery and organisation of care. [19].

There has been an international policy impetus to improve coordination for patients with rare conditions [7,11]. A review of 11 national policies for rare diseases showed that initiatives to improve coordination vary across countries [11]. For example, the introduction of centres of expertise, networks, databases and registries [11].

No reviews have focused on care coordination for rare conditions. Whilst there have been reviews of care coordination for chronic conditions conducted previously [15,18], there is a need to update reviews to include new evidence. This review will update knowledge of care coordination for common and rare chronic conditions. Given the paucity of reviews on rare conditions [20], this review will extend previous research by supplementing review findings with stakeholder consultations with patients, carers and healthcare providers who have experience of rare conditions. This will help to determine whether definitions and components of coordination are shared across all common and rare chronic conditions or whether some are specific to rare conditions. This will further our understanding of care coordination for rare conditions; given the lack of evidence that currently exists on care coordination for rare diseases.

This review of reviews seeks to extend previous knowledge by providing one of the first reviews of care coordination for rare conditions. To do this our review seeks to: (i) develop an updated definition of coordination of care for chronic conditions (rare and common), (ii) identify key components of care coordination for chronic conditions (rare and common), and categorise these according to their role in care coordination, and (iii) identify whether findings apply to rare conditions.

The overarching aim of this study was to provide a definition of coordinated care and identify components of coordinated care for rare diseases. To do this, we draw on literature from chronic conditions.

## Methods

A scoping review methodology was used. This manuscript followed reporting standards outlined in the ‘Preferred Reporting Items for Systematic reviews and Meta-Analyses (PRISMA) extension for Scoping Reviews (PRISMA-ScR)’ checklist [21].

We followed six recommended steps for conducting scoping reviews [21]. We 1) defined the research questions, 2) identified relevant studies, 3) selected reviews, 4) charted the data, 5) collated, summarised and reported the results and 6) consulted with stakeholders [22,23,24] (see Table 1).

**Table 1:** A description of our scoping review methods in relation to the six stages proposed by Arksey & O’Malley [22].

Scoping review stage	Description of our method

1) Defined research question	All co-authors developed three research questions: What does coordinated care mean? What are the components of coordinated care? Do definitions and components of care coordination identified in the literature (largely from common chronic conditions) apply to rare conditions?
2) Identified relevant studies	**Information sources:** – Nine electronic databases (MEDLINE, Scopus, CINAHL Plus, Web of Science, ProQuest Social Science, PubMed, Cochrane Database of systematic reviews, Database of abstracts of reviews of effects, and ProQuest Nursing and Allied Health) (1^st^author). – One reviewer (1^st^author) hand-searched key journals (BMJ Quality and Safety, Orphanet Journal of Rare Diseases, Journal of Health Services Research and Policy, Implementation Science). – Searched reference lists of included reviews (1^st^author). – Sent included reviews to five experts to identify any missing relevant reviews (one responded). **Search terms:** – Developed around the research questions. – Developed using the search terms used in a review (and the articles citing this review: McDonald et al. [15]), terminology used in coordination grey literature [2,5,7; 27,28,29] and peer reviewed articles [30,31,32] (by 1^st^ author). – Additional search terms identified through MEDLINE mapping function. – Search terms for reviews based on previous research [33]. – Search strategy reviewed by research team and subject librarian. – Search terms piloted and refined to check identification of key reviews. – Final search conducted (papers published from 2006, up until the date of the search in September 2018). **Eligibility criteria** (Developed and agreed within the wider research team): Focus on coordination of care within an intervention (Interventions that included both coordinated care and non-coordinated care interventions were excluded. To be inclusive, a range of terms for coordination were included). Focus on chronic or long-term health conditions, including ‘rare’, ‘ultra-rare’, undiagnosed and ‘non-rare’ (common) conditions (To take into account variations in definitions of chronic diseases and rare conditions, broad search terms were used). Provide a definition of coordinated care and information on the components of coordinated care. Review papers (all types of review included as long as a clear method was outlined. For example: narrative reviews, meta-analysis, systematic reviews and scoping reviews). Included health setting (Reviews which included articles that focus on other sectors were also included if they included health setting as well (e.g. social care). Reviews which focused on other sectors alone were excluded) Reviewed international research. Given that rare conditions affect patients all over the world [11], and that different countries have different healthcare systems and variations in how healthcare is delivered, it is important to learn how care is coordinated for both common and rare chronic conditions in different countries with different healthcare systems. Published after 2006 (This year was chosen to capture relevant major policy changes and to take into account a comprehensive review which included reviews prior to 2006 [15]). Written in English Published in peer-reviewed journals or grey literature
3) Selected reviews	– One reviewer (1^st^ author) conducted search – Guidelines developed around exclusion criteria – Reviewed in three stages: (i) titles, (ii) abstracts, (iii) full texts – Percentage independently screened by 2^nd^ researcher (2^nd^ author/TS) ° 40% of titles (n = 712). Agreement for different rounds of title screening ranged from: 60.1–78% ° 30% of abstracts (n = 226). Agreement for different rounds of abstract screening ranged from: 66.7–76% ° 5% of full texts (n = 24). Agreement: 73.9%. Also double screened additional full texts that were unclear (n = 14). – Researchers met to discuss decisions, resolve discrepancies and amend guidelines – One researcher (1st author) checked screening for consistency with amendments – Full-texts that were unclear retained until after data extraction when more information was available [34]
4) Charted data	– Developed a data charting form – One researcher (1^st^ author) used the form to chart data for all included reviews – Form included: review author, year of publication, review location, details of the programmes reviewed, scope of the review, aims of the review, type of review, outcome measures and important results in relation to the research questions and coordination of care (definitions of coordinated care and components of coordinated care). – Second researcher (2^nd^ author) extracted data from 10% of reviews identified in the initial electronic and hand search – Researchers met to discuss and resolve discrepancies [23]. – Prior to publication, all data extraction forms were rechecked to ensure the identification of components was comprehensive. Additional information was identified in less than 10% of reviews (n = 14).
5) Collated, summarised and reported results	– Thematic analysis used to develop definitions, identify and group components – 10% of components grouped independently by a second researcher (3^rd^ author) – Wider research team reviewed and agreed categorisation of components.
6) Consultation with stakeholders	**Sample:** – Three focus groups were conducted with adults aged 18 or over ° Two focus groups with patients or carers with rare conditions (one virtual focus group (PC1), one face-to-face focus group (PC2)) ° One focus group with healthcare professionals (HCPs) with expertise in rare conditions – Opportunity sampling through our charity partners **Procedure:** – During the focus groups discussed a summary of early findings from the review ° First focus group (FG-PC1) reviewed findings from 26 reviews ° Second focus group (FG-HCP) reviewed findings from 101 reviews ° Last focus group (FG-PC2) reviewed findings from 127 reviews (all reviews identified prior to expert and reference list searches) – Focus groups were audio-recorded, transcribed and fully anonymised **Analysis:** – Thematic analysis used to analyse the data – Two researchers inductively coded the three focus group transcripts (1^st^/4^th^ authors) – Findings discussed with research team and refined

As scoping reviews need to be rigorous and transparent, we followed a systematic approach [22]. Scoping review methodology was appropriate as defining coordinated care and identifying components of coordinated care for common and rare chronic conditions is a broad topic which requires accumulation of evidence using different study designs [22]. Additionally, scoping review methodology enabled a configurative approach (understanding and interpreting) which was appropriate for the purpose of the review [25,26].

An optional step of consulting with stakeholders through focus group methodology was included as there is a need to explore stakeholder views on whether findings from a scoping review on both common and rare chronic conditions also can be applied to rare conditions. This is consistent with research which suggests that stakeholder contributions enhances the usefulness of review findings (Oliver, 2001; as cited in 22].

### 1) Defined research questions

The research team developed three research questions (see Table 1).

### 2) Identified relevant studies

To identify relevant reviews, we searched electronic databases, hand-searched key journals and reference lists of included reviews, and asked experts to identify missing papers. See Table 1 for eligibility criteria, information sources and development of search terms.

Our search strategy included key words referring to coordinated care (including other terms for coordination, e.g. integration), components of coordinated care, the target population (including rare and chronic conditions), healthcare delivery and review methodology (See Supplementary file 1).

### 3) Selected reviews

Duplicate records were removed prior to screening. To facilitate screening, guidelines were developed around the eligibility criteria. Articles were reviewed against inclusion and exclusion criteria in three stages: (i) titles, (ii) abstracts, and (iii) full texts. All titles, abstracts and full texts were screened by one researcher (1st author) and a percentage of records were screened by an independent researcher (2^nd^ author/TS) [22]. Researchers met to discuss decisions, resolve discrepancies and amend guidelines (see Table 1).

### 4) Charted the data

A data extraction form was developed and used by one researcher (1^st^ author) to chart the data for all included reviews (see Table 1). To ensure consistency and thoroughness, a second researcher extracted data from 10% of the reviews (2^nd^ author). The researchers met to discuss and disagreements were resolved [22]. Data-extraction forms were also checked for comprehensiveness prior to publication.

Information on definitions and components were extracted from the whole review paper. To include as many components as possible, we extracted all components reported in review papers (including those reported from individual studies within the review).

### 5) Collated, summarised and reported results

Narrative analysis was used [35]. For each research question, findings were summarised using thematic analysis.

To develop a definition of coordinated care, individual definitions were coded inductively. These codes were grouped by one researcher (1^st^ author). Groups of codes were used to develop a preliminary definition (1^st^ author). The definition was reviewed and amended by the wider research team. Stakeholder consultation findings were then used to adapt this definition.

Components were coded and grouped by one researcher (1^st^ author). Examples of groups included: ‘Planning’, ‘Methods of coordination’, ‘Approaches of coordination’. A second researcher (3^rd^ author) double-coded 10% of components (excluding those identified through the expert search) into these groups. Disagreements were discussed and resolved by the two researchers. One researcher (1^st^ author) coded all remaining components according to rules agreed during this discussion. Groups of components were developed into themes and sub-themes. The number of reviews that reported each theme, sub-theme, and component was recorded. This helped to identify which components were more commonly used for chronic diseases.

**Figure 1:**
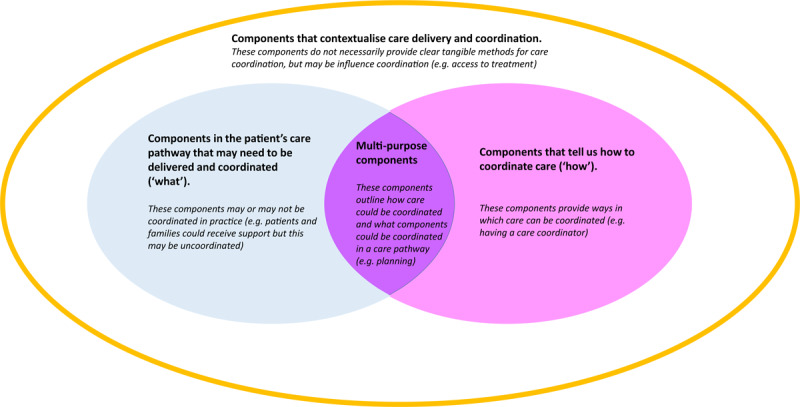
Categorisation of components of coordinated care.

To further develop our understanding of coordinated care, individual components were reviewed and categorised (by 1^st^ author) into four types of components using component descriptions from review papers. The four types of components (see Figure 1) were developed in discussions between the first and last author, and reviewed by the whole team. The wider research team reviewed and agreed the categorisation of components.

### 6) Consultation with stakeholders

Three focus groups (face-to-face or virtual) were conducted with patients/carers (aged 18+) affected by rare conditions and healthcare professionals who support patients with rare conditions (aged 18+). Stakeholder consultations concentrated on rare conditions in order to explore whether the individual definitions and components identified from the scoping review (which mainly covered common conditions) were applicable to rare conditions (see Table 1).

Participants were recruited through social media and charity partner organisations. To ensure a wide range of perspectives and experiences, we sampled participants using pre-specified eligibility criteria, including experience of care coordination, type of condition, age range and location).

Focus group participants were sent a summary of preliminary scoping review findings prior to the focus group, which included examples of individual definitions from review papers and examples of components. This summary was reviewed by a Public Patient Involvement expert for clarity, accessibility, and appropriate level of information.

Participants gave informed consent for participation. A structured topic guide was used to facilitate conversations (Supplementary file 2). This covered participants’ condition or role at work; their thoughts on definitions of coordinated care; their views on the scoping review findings (including the definitions and components); relevance to coordinated care for rare conditions; whether there were any components not identified and what components worked well and were difficult.

Focus groups were audio-recorded and transcribed by a professional transcription company. Transcripts were checked for accuracy and fully anonymised. Two researchers (1^st^/4^th^ authors) inductively coded the three focus group transcripts. Thematic analysis was used to analyse the data in relation to the three research questions [36]. Findings were discussed with the wider research team and refined.

## Results

### Review characteristics

**Figure 2:**
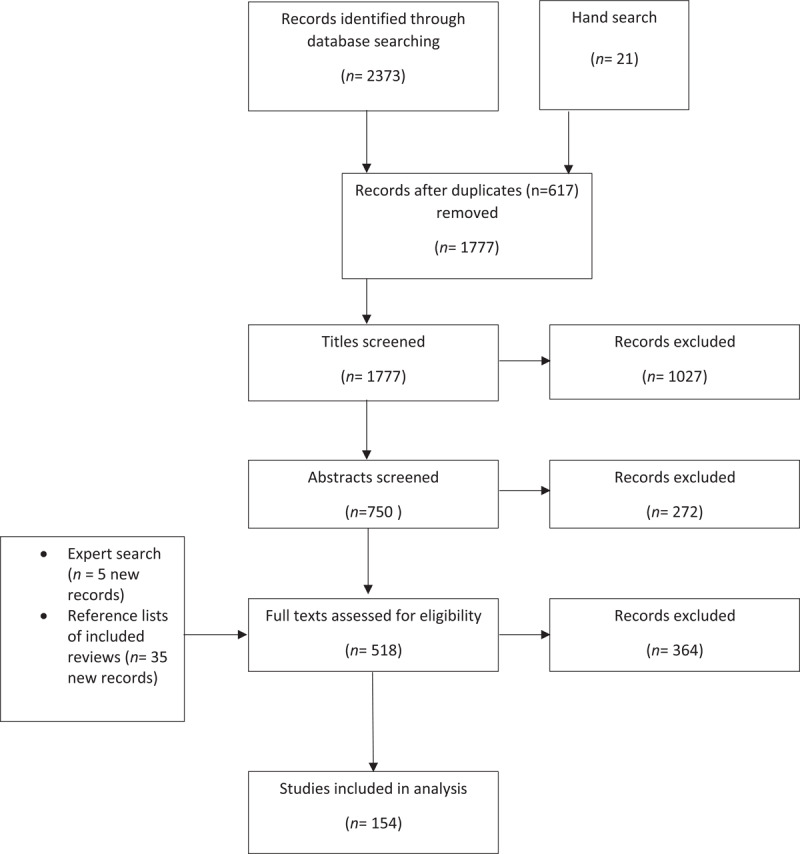
A flow chart showing the study selection process (based on Moher et al [52]; PRISMA).

One hundred and fifty-four reviews were included (for a list of reviews and their characteristics, see Supplementary files 3 and 4). Figure 2 outlines the review selection process.

The majority of reviews (n = 139) concerned care of common chronic conditions such as depression, anxiety, diabetes and heart failure. Three reviews concerned care for a single rare condition [37,38,39] and 12 included both rare and common chronic conditions [40,41,42,43,44,45,46,47,48,49,50,51]. Rare conditions included: Anorectal malformations, Guillain-Barré syndrome, Mayer-Rokitansky-Küster-Hauser syndrome, Sickle Cell disease, Juvenile Idiopathic Arthritis, Cystic Fibrosis, Spina bifida, Creutzfeldt-Jakob disease and Duchenne Muscular Dystrophy.

### Stakeholder consultation characteristics

Participant characteristics are shown in Table 2.

**Table 2:** Stakeholder consultation participant characteristics.

	Focus group			Total

	1	2	3	

Mode of delivery	Virtual	Face-to-face	Face-to-face	
Number of participants	7	4	6	17
Type of participant				
Patients	4	N/A	2	6
Parents/carers^1^	3	N/A	4	7
Healthcare professionals^2^	N/A	4	N/A	4
Gender				
Male	1	0	3	4
Female	6	4	3	13
Age				
29–59	6	N/A	3	9
60+	1	N/A	2	3
Not specified	0	N/A	1	1
Diagnosis				
One specific rare condition	4	N/A	3	7
Multiple chronic conditions (including at least one rare condition)	2	N/A	3	5
Undiagnosed	1	N/A	0	1
Number of regions^3^ represented	4	3	4	7

*Note*: ^1^ Parents and carers were included to capture views of caring for adults (n = 2) and children (n = 4) with rare, ultra-rare or undiagnosed conditions. ^2^ Job roles included consultants, nurses and a representative from a rare disease organisation (who had previous experience as a healthcare professional). In addition to their clinical role, one healthcare professional also worked for a rare disease organisation. ^3^ Regions refers to regions within the United Kingdom, including regions within England, in addition to Scotland, Wales and Northern Ireland. N/A = not applicable as patients/carers and healthcare professionals were asked different eligibility questions.

### 1. What does coordinated care mean in the context of rare conditions?

The scoping review identified many terms and definitions used to describe coordinated care. The most frequently used terms included: integrated care models/integrated care (n = 34, 22.1%), transition/care transition (n = 18, 11.7%), collaborative care (n = 15, 9.7%), transition from child to adult services (n = 15, 9.7%), disease management (n = 14, 9.1%), care coordination (n = 9, 9.5%) and case management/patient navigator (n = 8, 5.2%). See Supplementary file 5 for terms, number of reviews and example definitions.

Stakeholder consultation participants reported that these terms and definitions were relevant for rare conditions, emphasising the importance of communication, expertise and multidisciplinary teams for coordination. Healthcare professionals proposed that definitions need not specify a number of healthcare professionals and that transition is a component, but not a definition of coordination. In addition, stakeholder consultation findings highlighted the importance of care coordination being delivered equitably across geographical areas, individualisation, the importance of the whole family, and the need to coordinate care across a patient’s whole lifetime (including antenatal care through to bereavement care).

A definition of coordinated care for rare conditions was developed (see **Box 1**).

**Box 1: Definition of coordinated care for rare conditions (additions from stakeholder consultation findings in bold).**

*Coordination of care involves working together across multiple components and processes of care to enable everyone involved in a patient’s care (including a team of healthcare professionals, the patient and/or carer **and their family**) to avoid duplication and achieve shared outcomes, throughout a person’s whole life, across all parts of the health and care system, including*:

- Care from different healthcare services (e.g. different medical disciplines - medical, mental health, behavioural, health promotion)
- Care from different healthcare settings (including primary and secondary; community settings e.g. social care) and locations (e.g. rural/urban)
- Care across multiple conditions, or single conditions that affect multiple parts of the body
- The movement from one service, or setting, to another

*Coordination of care should be* ***family-centred***, *holistic (including a patient’s medical, psychosocial, educational and vocational needs), evidence-based*, ***with equal access to coordinated care irrespective of diagnosis, patient circumstances and geographical location***.

### 2. Components of coordinated care for rare conditions

Scoping review components were grouped into five themes, each with a number of sub-themes. Themes were: 1) Care pathway (components that related to the care pathway), 2) Approaches (components relating to care/coordination approaches), 3) Support (components relating to support), 4) Features (components relating to features of care), and 5) wider environment. Each theme had a number of sub-themes which each contained multiple components (see Supplementary file 6 for themes, sub-themes, individual components, the number of reviews that they were reported in and the type of components).

As the scoping review findings focus mainly on common chronic conditions, the stakeholder consultation findings were used to situate the relevance of components identified from the scoping review in the context of rare diseases. The next section integrates both the scoping review findings and the stakeholder consultation findings in order to highlight aspects of stakeholder consultation findings that supported, refuted or extended scoping review findings. Illustrative quotes are shown in Supplementary file 7.

**Figure 3:**
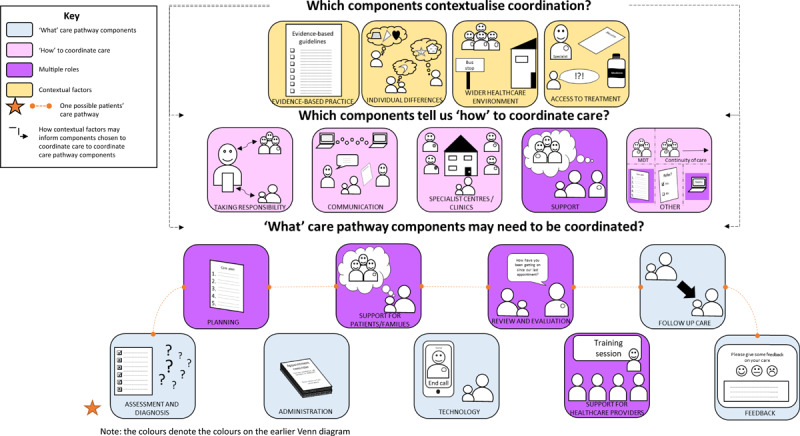
A diagram to outline a summary of components of care coordination, from scoping review and stakeholder consultation findings.

Many components were identified. Figure 3 provides an overview of scoping review and stakeholder consultation findings. The figure provides a summary of components within each of the four categories: components that may need to be coordinated inform how to coordinate care, have multiple roles and contextualise coordination. Each category is discussed in more detail below. Potential interactions between types of components are also highlighted. For example, the factors which contextualise coordination may influence which components may be needed to coordinate care. In turn, different types of ‘how’ components may be needed to coordinate different types of components in a patients’ care pathway.

#### ‘What’ care pathway components

Components that may need to be coordinated are related to administration, assessment and diagnosis, planning, review and evaluation, feedback, follow-up care and technology. The scoping review found that components relating to these aspects of care were frequently reported. For example: development of care plans (61.7%), follow-up care (52.6%), monitoring (51.3%), assessment of physical and mental health status (37.7%), reminders (26.6%), telecare (24%) and feedback for healthcare professionals (18.2%). Stakeholder consultation findings highlighted the importance of these components in the context of rare diseases. For example, assessment and diagnosis were identified as key elements for providing access to necessary care. While identified as important, participants reported not having care plans or regular reviews despite their potential to prevent crises and ensure ongoing provision of appropriate care.

The scoping review highlighted many support components for patients, carers and families which need to be coordinated. For example: education/skills training for patients (73.4%), self-management support (54.6%), psychological support for patients (50%) and support for carers (26%). Stakeholder consultation findings indicated that support for patients, carers and families with rare conditions was a crucial component of care including support for a range of needs (medical, psychological, practical, emotional and social), and support from various people (healthcare providers, peers, schools, patient support groups). Despite the importance of support, patients reported a lack of support and information provision for rare and undiagnosed conditions.

Finally, scoping review findings highlighted support required by healthcare professionals in providing care for patients with chronic conditions. Key support components included education (32.5%) and training (31.2%). Stakeholder consultation suggested that healthcare professionals working with patients with rare conditions needed support in accessing specialist knowledge, and addressing perceived fears, anxieties and negative attitudes towards treating rare conditions. However, those providers who had developed expertise were perceived to be equipped to provide quality care for people with rare conditions. This highlights the importance of training and education for care providers of people with rare conditions; including providers within healthcare, educational and voluntary sectors.

#### Components indicating ‘how’ care can be coordinated

Components outlining ‘how’ care can be coordinated are discussed below. There is potential overlap between some of these components and they are not mutually exclusive (e.g. ‘condition-specific’ centres or single visit approaches may use some of the other identified components in order to coordinate care).

##### Coordination through someone taking responsibility

Care could be coordinated using components relating to healthcare professionals, patients, and/or carers taking responsibility for coordinating care. Scoping review findings highlighted frequently reported components related to responsibility, including: coordination (e.g. collaboration, case management, disease management, an integrated approach) (70.8%) and responsibility for coordination by one healthcare provider (70.8%). Other components included patients’ coordinating their own treatments (16.9%).

Stakeholder consultation findings provide support for, and extend review findings by suggesting that for rare conditions, having someone take responsibility for coordinating care is key. However, as with scoping review findings, ‘who’ should take responsibility was more contested. Options included: healthcare professionals, patients or carers, administrative coordinators, departmental advocates, or GPs acting as gatekeepers for care for patients with rare conditions. Stakeholder consultation findings indicated that healthcare professionals as coordinators were perceived as helpful. Participants talked about a lack of formal care coordinator for many rare conditions and undiagnosed conditions. Similarly, patients and carers expressed concerns about whether individual coordinators could effectively coordinate everyone’s care due to limited capacity. The appropriateness of patients and/or carers as coordinators and what their role should be was unclear. Some patients/carers and healthcare professionals indicated that patients may be best placed to coordinate care whereas others emphasised negative implications and views against coordinating their own care. Patients and carers mostly wanted to be seen as care partners, with control over some but not all aspects. An administrative coordinator was also discussed as valuable and necessary by healthcare professionals, patients and carers. Findings therefore indicate that there may not be a single best approach when considering responsibility. Instead, a model of responsible coordinator(s) that best suit the whole family’s needs and situation should be negotiated during conversations about such responsibilities.

##### Coordination through specialist centres or joined up clinics

Care could be coordinated through specialist centres or joined up clinics. Scoping review findings indicated many different options for coordinating care through such centres, including: single visit approaches (40.3%), joint clinics or consultations (14.9%) and specialist or condition specific clinics (22.7%). Stakeholder consultation findings highlight that for rare conditions, specialist/condition-specific clinics were perceived as useful methods of coordination and many patients and carers reported attending specialised services. But, they are not available for all rare or undiagnosed conditions. Participants mostly reviewed clinics positively but also expressed views on: wanting to go to a clinic for some things but not others and clinics not being delivered to a standard. Clinics were thought to improve communication, and reduce traveling. Barriers to coordinating care through specialist centres included: needing funding, needing a diagnosis, needing ways to assess whether the centre is in fact a centre of excellence, needing space and the move from specialist to general clinics.

##### Coordination through communication

Care could be coordinated through differing methods of communication by healthcare professionals, patients and carers, including verbal and written communication and the use of technology. Many components identified in the scoping review related to verbal and written communication, including communication between providers and patients (57.1%), using and sharing documentation (46.8%), and team meetings to discuss coordination (76.6%).

These findings were supported by stakeholder consultation findings which indicated a lack of communication in practice for rare conditions. For example, findings indicated that documentation is often not shared with relevant care providers; resulting in a lack of care coordination for people with rare conditions. Patients reported acting as a medium for sharing documents between healthcare providers. This sometimes involved carrying large quantities of paperwork to appointments. Similarly, healthcare professionals were perceived by patients as not talking to each other; which resulted in message fatigue for patients and carers (repeating the same information multiple times). Using forms of identification for rare conditions (e.g. using Linnaean numbers, wearing pendants or using health passports) was perceived to be something that could facilitate coordination.

“¶114: you know, you should have access and all those people should speak to each other because it’s an interconnected condition and they don’t, in fact it’s quite hard to find a single person who knows.” (FG-PC1, patient)

Many components identified in the scoping review related to technology, including: electronic medical records (22.1%), communication systems/teleconferencing (22.7%), reminders for healthcare professionals (20.1%) and reminders for patients (7.1%). Similarly, stakeholder consultation findings indicated that patients, carers and healthcare providers thought that technology was a useful way of improving communication and therefore coordination for rare conditions. Participants reported many benefits including: facilitating communication between patients and providers outside and within the trust, sharing information and providing forms of patient identification. The need for joined up systems was highlighted as key for coordination. However, the NHS having many different systems that are not connected was perceived to limit coordination. Additional challenges included: data protection, people not having access to technology and financial constraints.

##### Coordination through support

The scoping review identified that care could be coordinated through different types of support for patients, families and healthcare professionals. Scoping review findings indicated that support may be provided from various sources, including healthcare professionals (including healthcare, social care and voluntary sector), family members and peers. Examples of components relating to support for patients which can be used to coordinate care, included: education and information/skills training (73.4%), general support for patients (68.2%), opportunities to familiarise with services (9.7%), and support for carers (26%). Similarly, scoping review findings indicate that some types of support for healthcare professionals may strengthen healthcare professionals’ capability to coordinate care, including training (31.2%), education (32.5%) and supervision (30.5%).

Stakeholder consultation findings highlighted that patient organisations and rare disease charities were perceived to be key in coordination, as they support patients and carers to develop expertise to take control over their condition. Providing patients, carers and healthcare professionals with the opportunity to familiarise themselves with services and development of clear expectations around coordination and self-management support was thought to help develop patients and carers expertise to coordinate and self-manage their care. This may be important given the potential role of patients and carers in care coordination (as highlighted previously). Opportunities included: involving patients in ward rounds and decision making. Stakeholder consultation findings also extend scoping review findings by highlighting the integral nature of support from schools for patients with rare conditions. Additionally, some participants reported a lack of support in some situations including for undiagnosed conditions and within private organisations (e.g. housing associations). A few patients and carers reported that access to support (e.g. hospice care) and support groups was limited for some rare and undiagnosed conditions.

##### Other coordination methods

Scoping review findings and stakeholder consultations indicated some other methods that can also be used to coordinate care, including multidisciplinary team approaches (76.6%), having the same individual care provider throughout (a key support of continuity of care) (14.9%), development of care plans (61.7%) and other planning components e.g. planning who is responsible for which aspects of care (11.7%), referrals (37%) and the use of registries (18.2%).

Stakeholder consultation indicated that continuity of healthcare providers was particularly important when multiple family members experienced the same symptoms, however findings indicated a lack of continuity in care for rare conditions. A lack of continuity was perceived as an advantage for patients with rare conditions by some healthcare professionals. For example, when transitioning from child to adult services and needing to take responsibility for self-managing the condition. The change in providers and the ‘transition’ component was perceived to be important in helping patients with rare conditions learn to advocate for themselves.

Stakeholder consultation participants reported a lack of care plans for rare conditions. Yet, stakeholder consultations indicated that care plans can be used to coordinate care in both every day and emergency situations. For example, planning can also help to coordinate care by facilitating team approaches and ensuring that teams are working towards shared goals; thus facilitating coordination.

#### Components that contextualise coordination

Scoping review findings demonstrated that evidence-based practice, individual differences and the wider healthcare environment may influence coordination. Components relating to these factors included: guideline-based treatment (37%), evidence-based treatment protocols (35.1%), individualised care (33.1%) and having a supportive environment for coordination (e.g. distance from treatment facilities/access to care) (31.2%). Stakeholder consultation findings indicated that to promote quality care and coordination, centralised and shared care pathways are needed. Yet findings indicated a lack of care pathways or defined standards for rare conditions or that treatments are not delivered consistently to standards (where standards are available).

Similarly, stakeholder consultation findings provided support for the importance of the wider healthcare environment by highlighting components relating to access to care that were relevant for rare conditions, including parents/patients fighting for access and barriers preventing access to records, results and medication. In terms of getting to healthcare appointments, participants reported having to travel to access care but that they were happy to do so if it meant they received expert care. One of the key issues preventing access to care in rare conditions was perceived to be limited availability of healthcare professionals with expertise in their condition. To take limited expertise in each condition into account, findings also indicated the need to succession plan by training more experts.

### 3. Application of definitions and components of care coordination from common chronic conditions to rare conditions

Overall, stakeholder consultation findings indicated that scoping review components were comprehensive and relevant for people with rare conditions. Participants expressed that rare conditions are mostly chronic conditions. As rare conditions and patients can vary substantially, participants felt that all components should be available even if they were not applicable to all patients’ individual situations.

“¶503: But P3 why are we living in a world where we have to pick one or two or three of these? Why can’t it be all of them?” (FG-PC1, patient)

Despite the perceived relevance of components, many participants described a lack of coordination for patients and families living with rare conditions. Participants described having to attend multiple appointments on different days, gaps and delays in sharing of documents and disagreements between healthcare professionals. Similarly, participants expressed concerns regarding coordination in emergency situations and the importance of participants taking control themselves in order to mitigate worries.

Coordinating care for rare and undiagnosed conditions may have further complexities that limit care and coordination. For example, difficulties diagnosing rare conditions. Diagnosis of rare conditions depends on factors such as a clinician’s knowledge and ability to recognise symptoms. Yet, developing condition-specific expertise for rare conditions is challenging given the small numbers of people diagnosed with each condition.

Despite many similarities between common and rare chronic conditions, participants expressed views that some components were missing or that need to be emphasised for rare conditions. These included: having someone to take responsibility for coordination; genome-based medicine/genetic screening; social support needs; counselling; and, antenatal and bereavement care. In addition, participants expressed views that more focus should be given to undiagnosed patients and families for many of the components.

“¶667: […] the whole of the NHS is, well, as [Patient charity] knows is going towards genome based medicine, so I think you need to recognise that.” (FG-PC2, carer)

## Discussion

### Key findings

Our definition indicated that coordinated care for people affected by rare conditions requires working together across multiple components and processes of care to ensure that everyone involved achieves shared outcomes across a person’s whole life throughout different parts of the health and care system. Our definition highlights that for rare conditions, coordinated care should be family-centred, evidence-based and equitable for all.

These review findings suggest that many of the key components and issues for coordinated care apply to both rare and common chronic conditions. However, stakeholder consultation findings highlighted additional components and context-specific issues that are relevant in the context of rare conditions.

### How findings relate to previous research and policy

Many terms and definitions were used to refer to care coordination, supporting previous research [15,53]. Our findings extend this research by developing a definition of coordination for rare conditions. Our definition supports previous research and the government’s rare disease strategy which highlight that care needs to be coordinated across boundaries, involve family members, be equitable and individualised [4,7].

A key challenge of coordination is that almost all aspects of care are relevant to coordination; making it difficult to distinguish between aspects of care and coordination components [19]. In this paper, we grouped components in relation to their roles, providing context for coordination components within the wider healthcare pathway and environment. We outlined four types of components, those that may need to be coordinated, inform how to coordinate care, have multiple roles, or that contextualise coordination. These categories overlap with those outlined in previous research [15,18], but provide clarity on ways in which different coordination components may be involved in complex care processes.

We identified far more reviews for common conditions than rare conditions, highlighting that little is currently known about coordination for rare conditions. Our findings extend previous research by highlighting similarities between definitions and components of coordination for both common and rare chronic conditions. Stakeholder consultation findings indicated that despite similarities in need, coordination for rare conditions may be less consistent; as components may not be delivered frequently in practice. This extends findings which indicate a lack of support [8], a lack of coordination, communication and a ‘postcode lottery’ approach to care [5] for people with rare conditions. Focus group findings indicated that many of the care components outlined in the review were not currently delivered effectively or consistently for people with rare conditions, let alone coordinated.

The difficulties associated with coordination for people with rare conditions in contrast to common chronic diseases may be attributed to complexities associated with rare conditions. For example, many rare conditions affect multiple parts of the body and many affect children. Therefore, care may need to be life-long, coordinated through transition periods, across multiple sectors (including schools) and must take family needs into account. In addition, diagnosis and expertise may be challenging due to fewer people living with each rare condition. This may mean that patients have to travel away from their area to access care. This may further complicate coordination. These complexities support research which suggests that more care coordination is needed in cases of greater system fragmentation, clinical complexity, and decreased patient capacity [15]. To account for this, findings indicate that we need an individualised approach to coordination for rare conditions. Some people may like to be more involved in coordination whereas other patients may need more coordination components in place. Stakeholder consultation findings proposed that components should be available to patients with rare and chronic conditions, and there could be a discussion between patients and healthcare professionals to determine appropriate needs for each individual.

Findings highlighted the role of patients and carers. Patient/carer roles included but were not limited to: taking responsibility for coordination, informing and updating healthcare professionals and fighting for access to care. For rare conditions, patients have a key role and in some cases are perceived as, or are responsible for coordination; supporting the burden of treatment theory [54]. Patients taking responsibility may be appropriate for some but not all patients and carers; particularly when conditions are complex, e.g. in the case of rare conditions. Methods of coordination which offer support to patients and carers (e.g. self-management support/opportunities to familiarise with services) may play an important role in involving patients/carers in coordination.

### Limitations

Given the paucity of literature on rare conditions, it was necessary to conduct a review for common and rare chronic conditions. Inclusive definitions were used for both coordination and chronic diseases. Despite our inclusive strategy, we are unlikely to have captured every relevant review. To identify as many studies as possible, we conducted a comprehensive search which included contacting experts and searching the reference lists of included reviews. Inclusive definitions for coordination and chronic diseases were used and reviews included research covering a range of countries.

Individual studies may be included in more than one of our included reviews. We based our analysis on the wording reported in the review papers, not individual studies. Therefore, we have not accounted for the possibility that authors may have reported components from individual studies in different ways. Components reported in published descriptions of interventions do not necessarily equate to the delivery of all components, therefore this review is limited to the components reported in the reviews.

The initial categorisation of components was based on researcher judgement. To limit individual subjectivity, the categorisation of components was reviewed and agreed within the wider research team. It is possible that other researchers may have categorised components differently.

This review focused on the identification of coordination components, rather than testing the effectiveness of coordination. Whilst we have outlined many different ways in which care can be coordinated; the effectiveness of combinations of, or individual components on relevant outcomes (such as reduced waiting times, better healthcare outcomes, better experience) is not known.

### Implications

Our findings may provide support for various international policy initiatives [7,9,11]. For example, our findings support the UK Strategy for Rare Diseases, as many of the components reported as necessary for coordination (e.g. diagnosis, care pathways, genetic testing, communication, care plans and training for healthcare professionals) were identified in our review [7]. Yet, our findings also highlight the complexity of delivering coordinated care by outlining many different options for coordinating care within practice. In particular, our findings emphasised the need for someone to take responsibility for coordination, as outlined in the NHS long term plan [9]. Our findings show that there are many ways in which responsibility could be managed. Whilst many different methods have been identified, some methods of coordination may be more complicated and multi-faceted than others. For example, condition-specific centres could facilitate other methods of coordination, such as communication and team meetings.

The components outlined in this review can be used to develop and evaluate existing and new models of coordination for common and rare chronic conditions. The contextual factors identified may need to be considered when developing models of care coordination for different groups.

### Future research

Evaluating the effectiveness of individual components was not within the scope of this review. The Coordinated Care of Rare Diseases project [55] aims to develop a taxonomy of coordinated care models for people with rare conditions. The taxonomy will build on these findings and enable researchers to test effectiveness and cost-effectiveness of different coordination models. Different care coordination approaches could also be evaluated in practice.

## Conclusions

Coordinated care requires working together across multiple components and processes of care to ensure everyone involved achieves shared outcomes across a person’s whole life throughout different parts of the health and care system. There are many components that could be delivered and coordinated as part of a patients’ care pathway, many ways to coordinate care and many factors which contextualise care coordination. Review findings indicate that many of the key components for coordinated care apply to rare and common chronic conditions. Stakeholder consultation findings highlighted additional components and context-specific issues that are relevant for rare conditions.

## Additional Files

The additional files for this article can be found as follows:

10.5334/ijic.5464.s1 Supplementary file 1. Search terms.

10.5334/ijic.5464.s2 Supplementary file 2. Topic guides.

10.5334/ijic.5464.s3 Supplementary file 3. References for reviews included in the scoping review.

10.5334/ijic.5464.s4 Supplementary file 4. Characteristics of included reviews.

10.5334/ijic.5464.s5 Supplementary file 5. Terms and definitions used.

10.5334/ijic.5464.s6 Supplementary file 6. Themes, sub-themes, components and type of components identified from reviews.

10.5334/ijic.5464.s7 Supplementary file 7. Example quotes from the stakeholder consultations for ‘what’, ‘how’ and ‘facilitating components’.
