# Supplementary material for: Defining Coordinated Care for People with Rare Conditions: A Scoping Review
Source: Int J Integr Care. 2020 Jun 25;20(2):14. doi: 10.5334/ijic.5464 (PMC7319081; doi:10.5334/ijic.5464)
Supplement: Supplementary file 1. — Search terms. [file ijic-20-2-5464-s1.pdf]

Supplementary file 1. Search terms

Database	Search number	Search
MEDLINE	1	(Care OR Service).ab,ti.
	2	(Co-ordination OR Coordinat* OR Co-ordinat* OR Coordination OR Collaborat* OR Collaborative OR Integrat* OR Integrated OR Shared OR Synchronised OR Synchronis* or Synchroniz* OR Synchronized OR Interdisciplinary OR Interdisciplin* OR Transitional OR Transition* OR cooperat* OR co-operat*).ab,ti.
	3	1 AND 2
	4	(Co-ordination of care OR Coordination of care OR Co-ordinat* of care OR Coordinat* of care OR Care co-ordination OR Care coordination OR Care co-ordinator OR Care coordinator OR Care coordinat* OR Care co-ordinat* OR Coordinated care OR Co-ordinated care OR Co-ordinated treatment OR Coordinated treatment OR Coordinating care OR Co-ordinating care OR Coordinat* care OR Co-ordinat* care OR Co-ordinat* treatment OR Coordinat* treatment OR Named coordinator OR Named coordinat* OR Named co-ordinator OR Named co-ordinat* OR Care advisor OR Patient navigator OR Care navigator OR Care organisation OR Care organisat* OR Care organization OR Care organizat* OR Care management OR Care manage* OR Case management OR Case manage* OR Disease management OR Disease manage* OR Condition management OR Condition manage* OR Organisation of patient care activities OR Organization of patient care activities OR Interprofessional network OR Interdisciplinary partnerships OR Integrated care OR Integrated care systems OR Co-management OR Co management OR Patient care planning OR Progressive patient care OR Multidisciplinary teams OR Multidisciplin* teams OR Multidisciplinary treatment OR Multidisciplin* treatment OR Multidisciplinary care OR Multidisciplin* care OR Collaboration OR Teamwork OR Model of care OR Continuity of care OR Continuity of patient care OR Care transitions OR Transition between care providers OR Participatory care OR Cross border cooperation OR Coordination across boundaries OR Co-ordination across boundaries OR Care pathway OR Care pathways OR Models of Care OR Care models OR Centres of excellence OR Specialist services OR Specialised services OR Speciali* services OR Specialist care OR Specialised care OR Speciali* care OR shared care OR transition* care OR transition of care OR transition* services OR transition* OR transfer of care OR patient care team OR patient transfer OR transition to adult care).ab,ti.
	5	3 OR 4
	6	(Co-ordination OR Coordination).ab,ti.
	7	(Component* OR Element* OR Activit* OR Feature* OR characteristic*).ab,ti.
	8	6 AND 7
	9	5 OR 8
	10	Condition* OR Disease* OR Disorder* OR illness* OR syndrome*
	11	Chronic OR Complex Chronic OR Long-term OR Long term OR Co-morbid* OR Co morbid OR comorbid OR Multi-morbid* OR multimorbid or multi morbid* OR Rare OR Very rare OR Ultra rare OR Ultra-rare OR Genetic OR undiagnosed OR undiagnosed genetic OR unknown OR unknown genetic OR without a name OR orphan
	12	10 AND 11
	13	9 AND 12
	14	Health OR Healthcare OR Health care OR Health-care
	15	Delivery OR Delivery, Integrated OR Integrated OR Delivery of OR Service*
	16	Delivery of healthcare, Integrated
	17/18	(14 AND 15) OR 16
	19	Intervention OR Evaluation
	20	19 AND 18
	21	13 AND 20

	22	((meta-analysis.pt or meta-analysis.tw or metanalysis.tw or ((review.pt or guideline.pt or consensus.ti or guideline*.ti or literature.ti or overview.ti or review.ti) and ((Cochrane.tw or Medline.tw or CINAHL.tw or (National.tw and Library.tw) or (handsearch*.tw or search*.tw or searching.tw)) and (hand.tw or manual.tw or electronic.tw or bibliographi*.tw or database* or (Cochrane.tw or Medline.tw or CINAHL.tw or (National.tw and Library.tw))))) or ((synthesis.ti or overview.ti or review.ti or survey.ti) and (systematic.ti or critical.ti or methodologic.ti or quantitative.ti or qualitative.ti or literature.ti or evidence.ti or evidence-based.ti))) not (case*.ti or report.ti or editorial.pt or comment.pt or letter.pt))
	23	21 and 22
	24	23+ filters > 2006
Scopus		(((((TITLE-ABS (care OR service) AND TITLE-ABS (co-ordination OR coordinat* OR co-ordinat* OR coordination OR collaborat* OR collaborative OR integrat* OR integrated OR shared OR synchronised OR synchronis* OR synchroniz* OR synchronized OR interdisciplinary OR interdisciplin* OR transitional OR transition* OR cooperat* OR co-operat*)) OR TITLE-ABS ("co-ordination of care" OR "coordination of care" OR "co-ordinat* of care" OR "coordinat* of care" OR "care co-ordination" OR "care coordination" OR "care co-ordinator" OR "care coordinator" OR "care coordinat*" OR "care co-ordinat*" OR "coordinated care" OR "co-ordinated care" OR "co-ordinated treatment" OR "coordinated treatment" OR "coordinating care" OR "co-ordinating care" OR "coordinat* care" OR "co-ordinat* care" OR "co-ordinat* treatment" OR "coordinat* treatment" OR "named coordinator" OR "named coordinat*" OR "named co-ordinator" OR "named coordinat*" OR "care advisor" OR "patient navigator" OR "care navigator" OR "care organisation" OR "care organisat*" OR "care organization" OR "care organizat*" OR "care management" OR "care manage*" OR "case management" OR "case manage*" OR "disease management" OR "disease manage*" OR "condition management" OR "condition manage*" OR "organisation of patient care activities" OR "organization of patient care activities" OR "interprofessional network" OR "interdisciplinary partnerships" OR "integrated care" OR "integrated care systems" OR co-management OR "co management" OR "patient care planning" OR "progressive patient care" OR "multidisciplinary teams" OR "multidisciplin* teams" OR "multidisciplinary treatment" OR "multidisciplin* treatment" OR "multidisciplinary care" OR "multidisciplin* care" OR collaboration OR teamwork OR "model of care" OR "continuity of care" OR "continuity of patient care" OR "care transitions" OR "transition between care providers" OR "participatory care" OR "cross border cooperation" OR "coordination across boundaries" OR "co-ordination across boundaries" OR "care pathway" OR "care pathways" OR "models of care" OR "care models" OR "centres of excellence" OR "specialist services" OR "specialised services" OR "speciali* services" OR "specialist care" OR "specialised care" OR "speciali* care" OR "shared care" OR "transition* care" OR "transition of care" OR "transition* services" OR transition* OR "transfer of care" OR "patient care team" OR "patient transfer" OR "transition to adult care")) OR (TITLE-ABS (co-ordination OR coordination) AND TITLE-ABS (component* OR element* OR activit* OR feature* OR characteristic*))) AND ((condition* OR disease* OR disorder* OR illness* OR syndrome*) AND (chronic OR complex AND chronic OR long-term OR long AND term OR co-morbid* OR "co morbid" OR comorbid OR multi-morbid* OR multimorbid OR "multi morbid*" OR rare OR "very rare" OR "ultra rare" OR ultra-rare OR genetic OR undiagnosed OR "undiagnosed genetic" OR unknown OR "unknown genetic" OR "without a name" OR orphan))) AND ((((health OR healthcare OR "health care" OR health-care) AND (delivery OR "delivery, integrated" OR integrated OR "delivery of service*")) OR ("delivery of healthcare, integrated")) AND (intervention OR evaluation))) AND ((meta-analysis OR meta-analysis OR metanalysis OR ((review OR guideline OR consensus OR guideline* OR literature OR overview OR review) AND ((cochrane OR medline OR cinahl OR (national AND library) OR (handsearch* OR search* OR searching)) AND (hand OR manual OR electronic OR bibliographi* OR database* OR (cochrane OR medline OR cinahl OR (national AND library))))) OR ((synthesis OR overview OR review OR survey) AND (systematic OR critical OR methodologic OR quantitative OR qualitative OR literature OR evidence OR evidence-based))) AND NOT (case* OR report OR editorial OR comment OR letter))
		Limits: English, human, 2006-2018 and review
CINAHL Plus	1	TI ((Care OR Service)) OR AB ((Care OR Service))
	2	TI (Co-ordination OR Coordinat* OR Co-ordinat* OR Coordination OR Collaborat* OR Collaborative OR Integrat* OR Integrated OR Shared OR Synchronised OR Synchronis* or Synchroniz* OR Synchronized OR Interdisciplinary OR Interdisciplin* OR Transitional OR Transition* OR cooperat* OR co-operat*) OR AB (Co-ordination OR Coordinat* OR Co-ordinat* OR Coordination OR Collaborat* OR Collaborative OR Integrat* OR Integrated OR Shared

		OR Synchronised OR Synchronis* or Synchroniz* OR Synchronized OR Interdisciplinary OR Interdisciplin* OR Transitional OR Transition* OR cooperat* OR co-operat*)
	3	S1 AND S2
	4	TI (“Co-ordination of care” OR “Coordination of care” OR “Co-ordinat* of care” OR “Coordinat* of care” OR “Care co-ordination” OR “Care coordination” OR “Care co-ordinator” OR “Care coordinator” OR “Care coordinat*” OR “Care co-ordinat*” OR “Coordinated care” OR “Co-ordinated care” OR “Co-ordinated treatment” OR “Coordinated treatment” OR “Coordinating care” OR “Co-ordinating care” OR “Coordinat* care” OR “Co-ordinat* care” OR “Co-ordinat* treatment” OR “Coordinat* treatment” OR “Named coordinator” OR “Named coordinat*” OR “Named co-ordinator” OR “Named co-ordinat*” OR “Care advisor” OR “Patient navigator” OR “Care navigator” OR “Care organisation” OR “Care organisat*” OR “Care organization” OR “Care organizat*” OR “Care management” OR “Care manage*” OR “Case management” OR “Case manage*” OR “Disease management” OR “Disease manage*” OR “Condition management” OR “Condition manage*” OR “Organisation of patient care activities” OR “Organization of patient care activities” OR “Interprofessional network” OR “Interdisciplinary partnerships” OR “Integrated care” OR “Integrated care systems” OR Co-management OR “Co management” OR “Patient care planning” OR “Progressive patient care” OR “Multidisciplinary teams” OR “Multidisciplin* teams” OR “Multidisciplinary treatment” OR “Multidisciplin* treatment” OR “Multidisciplinary care” OR “Multidisciplin* care” OR Collaboration OR Teamwork OR “Model of care” OR “Continuity of care” OR “Continuity of patient care” OR “Care transitions” OR “Transition between care providers” OR “Participatory care” OR “Cross border cooperation” OR “Coordination across boundaries” OR “Co-ordination across boundaries” OR “Care pathway” OR “Care pathways” OR “Models of Care” OR “Care models” OR “Centres of excellence” OR “Specialist services” OR “Specialised services” OR “Speciali* services” OR “Specialist care” OR “Specialised care” OR “Speciali* care” OR “shared care” OR “transition* care” OR “transition of care” OR “transition* services” OR transition* OR “transfer of care” OR “patient care team” OR “patient transfer” OR “transition to adult care”) OR AB (“Co-ordination of care” OR “Coordination of care” OR “Co-ordinat* of care” OR “Coordinat* of care” OR “Care co-ordination” OR “Care coordination” OR “Care co-ordinator” OR “Care coordinator” OR “Care coordinat*” OR “Care co-ordinat*” OR “Coordinated care” OR “Co-ordinated care” OR “Co-ordinated treatment” OR “Coordinated treatment” OR “Coordinating care” OR “Co-ordinating care” OR “Coordinat* care” OR “Co-ordinat* care” OR “Co-ordinat* treatment” OR “Coordinat* treatment” OR “Named coordinator” OR “Named coordinat*” OR “Named co-ordinator” OR “Named co-ordinat*” OR “Care advisor” OR “Patient navigator” OR “Care navigator” OR “Care organisation” OR “Care organisat*” OR “Care organization” OR “Care organizat*” OR “Care management” OR “Care manage*” OR “Case management” OR “Case manage*” OR “Disease management” OR “Disease manage*” OR “Condition management” OR “Condition manage*” OR “Organisation of patient care activities” OR “Organization of patient care activities” OR “Interprofessional network” OR “Interdisciplinary partnerships” OR “Integrated care” OR “Integrated care systems” OR Co-management OR “Co management” OR “Patient care planning” OR “Progressive patient care” OR “Multidisciplinary teams” OR “Multidisciplin* teams” OR “Multidisciplinary treatment” OR “Multidisciplin* treatment” OR “Multidisciplinary care” OR “Multidisciplin* care” OR Collaboration OR Teamwork OR “Model of care” OR “Continuity of care” OR “Continuity of patient care” OR “Care transitions” OR “Transition between care providers” OR “Participatory care” OR “Cross border cooperation” OR “Coordination across boundaries” OR “Co-ordination across boundaries” OR “Care pathway” OR “Care pathways” OR “Models of Care” OR “Care models” OR “Centres of excellence” OR “Specialist services” OR “Specialised services” OR “Speciali* services” OR “Specialist care” OR “Specialised care” OR “Speciali* care” OR “shared care” OR “transition* care” OR “transition of care” OR “transition* services” OR transition* OR “transfer of care” OR “patient care team” OR “patient transfer” OR “transition to adult care”)
	5	S3 or S4
	6	TI (Co-ordination OR Coordination) OR AB (Co-ordination OR Coordination)
	7	TI(Component* OR Element* OR Activit* OR Feature* OR characteristic*) OR AB (Component* OR Element* OR Activit* OR Feature* OR characteristic*)
	8	S6 AND S7
	9	S5 OR S8
	10	Condition* OR Disease* OR Disorder* OR illness* OR syndrome*
	11	Chronic OR Complex Chronic OR Long-term OR Long term OR Co-morbid* OR “Co morbid” OR comorbid OR Multi-morbid* OR multimorbid or “multi

		morbid*” OR Rare OR “Very rare” OR “Ultra rare” OR Ultra-rare OR Genetic OR undiagnosed OR “undiagnosed genetic” OR unknown OR “unknown genetic” OR “without a name” OR orphan
	12	S10 AND S11
	13	S9 AND S12
	14	Health OR Healthcare OR “Health care” OR Health-care
	15	Delivery OR “Delivery, Integrated” OR Integrated OR Delivery of OR Service*
	16	“delivery of health care, integrated”
	17	S14 AND S15
	18	S16 OR S17
	19	Intervention OR evaluation
	20	S18 AND S19
	21	S13 AND S20
	22	PT meta-analysis OR TX meta-analysis OR TX metanalysis
	23	(TX Cochrane OR TX Medline OR TX CINAHL) OR (TX National AND TX library)
	24	TX handsearch* OR TX search* OR TX searching
	25	TX hand OR TX manual OR TX electronic OR TX bibliographi* OR database
	26	S23 OR S25
	27	S24 AND S26
	28	PT review OR PT guideline OR TI consensus OR TI guideline* OR TI literature OR TI overview OR TI review
	29	S23 OR S27
	30	S28 AND S29
	31	(TI synthesis OR TI overview OR TI review OR TI survey) AND (TI systematic OR TI critical OR TI methodologic OR TI quantitative OR TI qualitative OR TI literature OR TI evidence OR TI evidence-based)
	32	S22 OR S30 OR S31
	33	(S22 OR S30 OR S31) NOT (TI case* OR TI report OR PT editorial OR PT comment OR PT letter)
	34	S21 AND S33
	35	**add filters to #34 here***
Web of Science	1	TS=(care "OR" service)
	2	TS=(Co-ordination OR Coordinat* OR Co-ordinat* OR Coordination OR Collaborat* OR Collaborative OR Integrat* OR Integrated OR Shared OR Synchronised OR Synchronis* or Synchroniz* OR Synchronized OR Interdisciplinary OR Interdisciplin* OR Transitional OR Transition* OR cooperat* OR co-operat*)
	3	#2 AND #1
	4	TS=(Co-ordination of care OR Coordination of care OR Co-ordinat* of care OR Coordinat* of care OR Care co-ordination OR Care coordination OR Care co-ordinator OR Care coordinator OR Care coordinat* OR Care co-ordinat* OR Coordinated care OR Co-ordinated care OR Co-ordinated treatment OR Coordinated treatment OR Coordinating care OR Co-ordinating care OR Coordinat* care OR Co-ordinat* care OR Co-ordinat* treatment OR Coordinat* treatment OR Named coordinator OR Named coordinat* OR Named co-ordinator OR Named co-ordinat* OR Care advisor OR Patient navigator OR Care navigator OR Care organisation OR Care organisat* OR Care organization OR Care organizat* OR Care management OR Care manage* OR Case management OR Case manage* OR Disease management OR Disease manage* OR Condition management OR Condition manage* OR Organisation of

		patient care activities OR Organization of patient care activities OR Interprofessional network OR Interdisciplinary partnerships OR Integrated care OR Integrated care systems OR Co-management OR Co management OR Patient care planning OR Progressive patient care OR Multidisciplinary teams OR Multidisciplin* teams OR Multidisciplinary treatment OR Multidisciplin* treatment OR Multidisciplinary care OR Multidisciplin* care OR Collaboration OR Teamwork OR Model of care OR Continuity of care OR Continuity of patient care OR Care transitions OR Transition between care providers OR Participatory care OR Cross border cooperation OR Coordination across boundaries OR Co-ordination across boundaries OR Care pathway OR Care pathways OR Models of Care OR Care models OR Centres of excellence OR Specialist services OR Specialised services OR Speciali* services OR Specialist care OR Specialised care OR Speciali* care OR shared care OR transition* care OR transition of care OR transition* services OR transition* OR transfer of care OR patient care team OR patient transfer OR transition to adult care)
	5	#4 OR #3
	6	TS=(Co-ordination OR Coordination)
	7	TS=(Component* OR Element* OR Activit* OR Feature* OR characteristic*)
	8	#7 AND #6
	9	#8 OR #5
	10	TS=(Condition* OR Disease* OR Disorder* OR illness* OR syndrome*)
	11	TS=(Chronic OR Complex Chronic OR Long-term OR Long term OR Co-morbid* OR Co morbid OR comorbid OR Multi-morbid* OR multimorbid or multi morbid* OR Rare OR Very rare OR Ultra rare OR Ultra-rare OR Genetic OR undiagnosed OR undiagnosed genetic OR unknown OR unknown genetic OR without a name OR orphan)
	12	#11 AND #10
	13	#12 AND #9
	14	TS=(Health OR Healthcare OR Health care OR Health-care)
	15	TS=(Delivery OR Delivery, Integrated OR Integrated OR Delivery of OR Service*)
	16	TS=(Delivery of healthcare, Integrated)
	17	#14 AND #15
	18	#16 OR #17
	19	TS=(Intervention OR Evaluation)
	20	#18 AND #19
	21	#20 AND #13
	22	TS=(meta-analysis OR meta-analysis OR metanalysis)
	23	TS=((Cochrane OR Medline OR CINAHL) OR (National AND Library))
	24	TS=(handsearch* OR search* OR searching)
	25	TS=(hand OR manual OR electronic OR bibliographi* OR database*) OR #23
	26	#25 AND #24
	27	TS=(review OR guideline)

	28	TI=(consensus OR guideline* OR literature OR overview OR review)
	29	#28 OR #27
	30	#26 OR #23
	31	#29 AND #30
	32	TI=((synthesis OR overview OR review OR survey) AND (systematic OR critical OR methodologic OR quantitative OR qualitative OR literature OR evidence OR evidence-based))
	33	#32 OR #31 OR #22
	34	TI=(case* OR report)
	35	TS=(editorial OR comment OR letter)
	36	#34 OR #35
	37	#33 NOT #36
	38	#21 AND #37
	39	(#38) ***english and date**
PubMed	1 (title/abstract)	(Care OR Service) AND (Co-ordination OR Coordinat* OR Co-ordinat* OR Coordination OR Collaborat* OR Collaborative OR Integrat* OR Integrated OR Shared OR Synchronised OR Synchronis* or Synchroniz* OR Synchronized OR Interdisciplinary OR Interdisciplin* OR Transitional OR Transition* OR cooperat* OR co-operat*)
	2 (title/abstract)	“Co-ordination of care” OR “Coordination of care” OR “Co-ordinat* of care” OR “Coordinat* of care” OR “Care co-ordination” OR “Care coordination” OR “Care co-ordinator” OR “Care coordinator” OR “Care coordinat*” OR “Care co-ordinat*” OR “Coordinated care” OR “Co-ordinated care” OR “Co-ordinated treatment” OR “Coordinated treatment” OR “Coordinating care” OR “Co-ordinating care” OR “Coordinat* care” OR “Co-ordinat* care” OR “Co-ordinat* treatment” OR “Coordinat* treatment” OR “Named coordinator” OR “Named coordinat*” OR “Named co-ordinator” OR “Named co-ordinat*” OR “Care advisor” OR “Patient navigator” OR “Care navigator” OR “Care organisation” OR “Care organisat*” OR “Care organization” OR “Care organizat*” OR “Care management” OR “Care manage*” OR “Case management” OR “Case manage*” OR “Disease management” OR “Disease manage*” OR “Condition management” OR “Condition manage*” OR “Organisation of patient care activities” OR “Organization of patient care activities” OR “Interprofessional network” OR “Interdisciplinary partnerships” OR “Integrated care” OR “Integrated care systems” OR Co-management OR “Co management” OR “Patient care planning” OR “Progressive patient care” OR “Multidisciplinary teams” OR “Multidisciplin* teams” OR “Multidisciplinary treatment” OR “Multidisciplin* treatment” OR “Multidisciplinary care” OR “Multidisciplin* care” OR Collaboration OR Teamwork OR “Model of care” OR “Continuity of care” OR “Continuity of patient care” OR “Care transitions” OR “Transition between care providers” OR “Participatory care” OR “Cross border cooperation” OR “Coordination across boundaries” OR “Co-ordination across boundaries” OR “Care pathway” OR “Care pathways” OR “Models of Care” OR “Care models” OR “Centres of excellence” OR “Specialist services” OR “Specialised services” OR “Speciali* services” OR “Specialist care” OR “Specialised care” OR “Speciali* care” OR “shared care” OR “transition* care” OR “transition of care” OR “transition* services” OR transition* OR “transfer of care” OR “patient care team” OR “patient transfer” OR “transition to adult care”
	3 (title/abstract)	(Co-ordination OR Coordination) AND (Component* OR Element* OR Activit* OR Feature* OR characteristic*)
	4	1 OR 2 OR 3
	5	Condition* OR Disease* OR Disorder* OR illness* OR syndrome*
	6	Chronic OR Complex Chronic OR Long-term OR Long term OR Co-morbid* OR “Co morbid” OR comorbid OR Multi-morbid* OR multimorbid or “multi

		morbid*” OR Rare OR “Very rare” OR “Ultra rare” OR Ultra-rare OR Genetic OR undiagnosed OR “undiagnosed genetic” OR unknown OR “unknown genetic” OR “without a name” OR orphan
	7	5 AND 6
	8	4 AND 7
	9	Health OR Healthcare OR “Health care” OR Health-care
	10	Delivery OR “Delivery, Integrated” OR Integrated OR Delivery of OR Service*
	11	“Delivery of healthcare, Integrated”
	12/13	(9 AND 10) OR 11
	14	Intervention OR Evaluation
	15	13 AND 14
	16	8 AND 15
	17	((meta-analysis [pt] OR meta-analysis [tw] OR metanalysis [tw]) OR ((review [pt] OR guideline [pt] OR consensus [ti] OR guideline* [ti] OR literature [ti] OR overview [ti] OR review [ti]) AND ((Cochrane [tw] OR Medline [tw] OR CINAHL [tw] OR (National [tw] AND Library [tw])) OR (handsearch* [tw] OR search* [tw] OR searching [tw]) AND (hand [tw] OR manual [tw] OR electronic [tw] OR bibliographi* [tw] OR database* OR (Cochrane [tw] OR Medline [tw] OR CINAHL [tw] OR (National [tw] AND Library [tw]))))) OR ((synthesis [ti] OR overview [ti] OR review [ti] OR survey [ti]) AND (systematic [ti] OR critical [ti] OR methodologic [ti] OR quantitative [ti] OR qualitative [ti] OR literature [ti] OR evidence [ti] OR evidence-based [ti]))) BUTNOT (case* [ti] OR report [ti] OR editorial [pt] OR comment [pt] OR letter [pt])
	18	16 AND 17
	19	18 + filters > 2006, peer reviewed, English
Cochrane Database of systematic reviews	1	(Care OR Service):ti,ab
	2	(Co-ordination OR Coordinat* OR Co-ordinat* OR Coordination OR Collaborat* OR Collaborative OR Integrat* OR Integrated OR Shared OR Synchronised OR Synchronis* or Synchroniz* OR Synchronized OR Interdisciplinary OR Interdisciplin* OR Transitional OR Transition* OR cooperat* OR co-operat*):ti,ab
	3	#1 AND #2
	4	(“Co-ordination of care” OR “Coordination of care” OR “Co-ordinat* of care” OR “Coordinat* of care” OR “Care co-ordination” OR “Care coordination” OR “Care co-ordinator” OR “Care coordinator” OR “Care coordinat*” OR “Care co-ordinat*” OR “Coordinated care” OR “Co-ordinated care” OR “Co-ordinated treatment” OR “Coordinated treatment” OR “Coordinating care” OR “Co-ordinating care” OR “Coordinat* care” OR “Co-ordinat* care” OR “Co-ordinat* treatment” OR “Coordinat* treatment” OR “Named coordinator” OR “Named coordinat*” OR “Named co-ordinator” OR “Named co-ordinat*” OR “Care advisor” OR “Patient navigator” OR “Care navigator” OR “Care organisation” OR “Care organisat*” OR “Care organization” OR “Care organizat*” OR “Care management” OR “Care manage*” OR “Case management” OR “Case manage*” OR “Disease management” OR “Disease manage*” OR “Condition management” OR “Condition manage*” OR “Organisation of patient care activities” OR “Organization of patient care activities” OR “Interprofessional network” OR “Interdisciplinary partnerships” OR “Integrated care” OR “Integrated care systems” OR Co-management OR “Co management” OR “Patient care planning” OR “Progressive patient care” OR “Multidisciplinary teams” OR “Multidisciplin* teams” OR “Multidisciplinary treatment” OR “Multidisciplin* treatment” OR “Multidisciplinary care” OR “Multidisciplin* care” OR Collaboration OR Teamwork OR “Model of care” OR “Continuity of care” OR “Continuity of patient care” OR “Care transitions” OR “Transition between care providers” OR “Participatory care” OR “Cross border cooperation” OR “Coordination across boundaries” OR “Co-ordination across boundaries” OR “Care pathway” OR “Care pathways” OR “Models of Care” OR “Care models” OR “Centres of excellence” OR “Specialist services” OR “Specialised services” OR “Speciali* services” OR “Specialist care” OR “Specialised care” OR “Speciali* care” OR “shared care” OR “transition* care” OR “transition of care” OR “transition* services” OR transition* OR “transfer of care” OR “patient care team” OR “patient transfer” OR “transition to adult care”):ti,ab
	5	#3 OR #4
	6	(Co-ordination OR Coordination):ti,ab

	7	(Component* OR Element* OR Activit* OR Feature* OR characteristic*):ti,ab
	8	#6 AND #7
	9	#5 OR #8
	10	Condition* OR Disease* OR Disorder* OR illness* OR syndrome*
	11	Chronic OR Complex Chronic OR Long-term OR Long term OR Co-morbid* OR “Co morbid” OR comorbid OR Multi-morbid* OR multimorbid or “multi morbid*” OR Rare OR “Very rare” OR “Ultra rare” OR Ultra-rare OR Genetic OR undiagnosed OR “undiagnosed genetic” OR unknown OR “unknown genetic” OR “without a name” OR orphan
	12	#10 AND #11
	13	#9 AND #12
	14	Health OR Healthcare OR “Health care” OR Health-care
	15	Delivery OR “Delivery, Integrated” OR Integrated OR Delivery of OR Service*
	16	“Delivery of healthcare, Integrated”
	17	#14 AND #15
	18	#17 OR #16
	19	Intervention or evaluation
	20	#18 AND #19
	21	#13 AND #20
	22	(meta-analysis):pt OR (meta-analysis OR metanalysis):ti
	23	(Cochrane OR Medline OR CINAHL) OR (National AND Library)
	24	(search* OR searching OR handsearch*)
	25	(hand OR manual OR electronic OR bibliographi* OR database*)
	26	#24 AND (#25 OR #23)
	27	(review OR guideline):pt AND (Consensus OR guideline* OR literature OR overview OR review):ti
	28	#27 AND (#23 OR #26)
	29	(synthesis OR overview OR review OR survey):ti AND (Systematic OR critical OR methodological OR quantitative OR qualitative OR literature OR evidence OR evidence-based):ti
	30	#22 OR #28 OR #29
	31	(case* OR report):ti OR (editorial OR comment OR letter):pt
	32	#30 NOT #31
	33	#21 AND #32
Database of abstracts of reviews of effects – cannot find this database to search		
Nursing and	1	ti((Care OR Service) AND (Co-ordination OR Coordinat* OR Co-ordinat* OR Coordination OR Collaborat* OR Collaborative OR Integrat* OR Integrated OR

Allied Health & Social sciences (ProQuest) – 2 databases		Shared OR Synchronised OR Synchronis* or Synchroniz* OR Synchronized OR Interdisciplinary OR Interdisciplin* OR Transitional OR Transition* OR cooperat* OR co-operat*)) OR ab((Care OR Service) AND (Co-ordination OR Coordinat* OR Co-ordinat* OR Coordination OR Collaborat* OR Collaborative OR Integrat* OR Integrated OR Shared OR Synchronised OR Synchronis* or Synchroniz* OR Synchronized OR Interdisciplinary OR Interdisciplin* OR Transitional OR Transition* OR cooperat* OR co-operat*))
	2	ti("Co-ordination of care" OR "Coordination of care" OR "Co-ordinat* of care" OR "Coordinat* of care" OR "Care co-ordination" OR "Care coordination" OR "Care co-ordinator" OR "Care coordinator" OR "Care coordinat*" OR "Care co-ordinat*" OR "Coordinated care" OR "Co-ordinated care" OR "Co-ordinated treatment" OR "Coordinated treatment" OR "Coordinating care" OR "Co-ordinating care" OR "Coordinat* care" OR "Co-ordinat* care" OR "Co-ordinat* treatment" OR "Coordinat* treatment" OR "Named coordinator" OR "Named coordinat*" OR "Named co-ordinator" OR "Named co-ordinat*" OR "Care advisor" OR "Patient navigator" OR "Care navigator" OR "Care organisation" OR "Care organisat*" OR "Care organization" OR "Care organizat*" OR "Care management" OR "Care manage*" OR "Case management" OR "Case manage*" OR "Disease management" OR "Disease manage*" OR "Condition management" OR "Condition manage*" OR "Organisation of patient care activities" OR "Organization of patient care activities" OR "Interprofessional network" OR "Interdisciplinary partnerships" OR "Integrated care" OR "Integrated care systems" OR "Co-management" OR "Co management" OR "Patient care planning" OR "Progressive patient care" OR "Multidisciplinary teams" OR "Multidisciplin* teams" OR "Multidisciplinary treatment" OR "Multidisciplin* treatment" OR "Multidisciplinary care" OR "Multidisciplin* care" OR Collaboration OR Teamwork OR "Model of care" OR "Continuity of care" OR "Continuity of patient care" OR "Care transitions" OR "Transition between care providers" OR "Participatory care" OR "Cross border cooperation" OR "Coordination across boundaries" OR "Co-ordination across boundaries" OR "Care pathway" OR "Care pathways" OR "Models of Care" OR "Care models" OR "Centres of excellence" OR "Specialist services" OR "Specialised services" OR "Speciali* services" OR "Specialist care" OR "Specialised care" OR "Speciali* care" OR "shared care" OR "transition* care" OR "transition of care" OR "transition* services" OR transition* OR "transfer of care" OR "patient care team" OR "patient transfer" OR "transition to adult care") OR ab("Co-ordination of care" OR "Coordination of care" OR "Co-ordinat* of care" OR "Coordinat* of care" OR "Care co-ordination" OR "Care coordination" OR "Care co-ordinator" OR "Care coordinator" OR "Care coordinat*" OR "Care co-ordinat*" OR "Coordinated care" OR "Co-ordinated care" OR "Co-ordinated treatment" OR "Coordinated treatment" OR "Coordinating care" OR "Co-ordinating care" OR "Coordinat* care" OR "Co-ordinat* care" OR "Co-ordinat* treatment" OR "Coordinat* treatment" OR "Named coordinator" OR "Named coordinat*" OR "Named co-ordinator" OR "Named co-ordinat*" OR "Care advisor" OR "Patient navigator" OR "Care navigator" OR "Care organisation" OR "Care organisat*" OR "Care organization" OR "Care organizat*" OR "Care management" OR "Care manage*" OR "Case management" OR "Case manage*" OR "Disease management" OR "Disease manage*" OR "Condition management" OR "Condition manage*" OR "Organisation of patient care activities" OR "Organization of patient care activities" OR "Interprofessional network" OR "Interdisciplinary partnerships" OR "Integrated care" OR "Integrated care systems" OR "Co-management" OR "Co management" OR "Patient care planning" OR "Progressive patient care" OR "Multidisciplinary teams" OR "Multidisciplin* teams" OR "Multidisciplinary treatment" OR "Multidisciplin* treatment" OR "Multidisciplinary care" OR "Multidisciplin* care" OR Collaboration OR Teamwork OR "Model of care" OR "Continuity of care" OR "Continuity of patient care" OR "Care transitions" OR "Transition between care providers" OR "Participatory care" OR "Cross border cooperation" OR "Coordination across boundaries" OR "Co-ordination across boundaries" OR "Care pathway" OR "Care pathways" OR "Models of Care" OR "Care models" OR "Centres of excellence" OR "Specialist services" OR "Specialised services" OR "Speciali* services" OR "Specialist care" OR "Specialised care" OR "Speciali* care" OR "shared care" OR "transition* care" OR "transition of care" OR "transition* services" OR transition* OR "transfer of care" OR "patient care team" OR "patient transfer" OR "transition to adult care")
	3	1 OR 2
	4	ti((Co-ordination OR Coordination) AND (Component* OR Element* OR Activit* OR Feature* OR characteristic*)) OR ab((Co-ordination OR Coordination) AND (Component* OR Element* OR Activit* OR Feature* OR characteristic*))
	5	3 OR 4
	6	Condition* OR Disease* OR Disorder* OR illness* OR syndrome*
	7	Chronic OR Complex Chronic OR Long-term OR Long term OR Co-morbid* OR “Co morbid” OR comorbid OR Multi-morbid* OR multimorbid or “multi

		morbid*" OR Rare OR "Very rare" OR "Ultra rare" OR Ultra-rare OR Genetic OR undiagnosed OR "undiagnosed genetic" OR unknown OR "unknown genetic" OR "without a name" OR orphan
	8	5 AND 6
	9	4 AND 9
	10	((Health OR Healthcare OR "Health care" OR Health-care) and (Delivery OR "Delivery, Integrated" OR Integrated OR Delivery of OR Service*)) OR ("Delivery of healthcare, Integrated")
	11	Intervention OR Evaluation
	12	10 AND 11
	13	9 AND 11
	14	((meta-analysis OR ft(meta-analysis OR metanalysis)) OR (((review OR guideline) OR ti(consensus OR guideline* OR literature OR overview OR review)) AND ((ft(Cochrane OR Medline OR CINAHL) OR ft(National AND Library)) OR (ft(handsearch* OR search* OR searching) AND ((ft(Cochrane OR Medline OR CINAHL) OR ft(National AND Library)) OR ft(hand OR manual OR electronic OR bibliographi* OR database*))))) OR (ti(synthesis OR overview OR review OR survey) AND ti(systematic OR critical OR methodologic OR quantitative OR qualitative OR literature OR evidence OR evidence-based))) NOT (ti(case* OR report) OR (editorial OR comment OR letter))
	15	13 AND 14
		Limits: English, human, 2006-2018 and possibly review
