# Supplementary material for: Defining Coordinated Care for People with Rare Conditions: A Scoping Review
Source: Int J Integr Care. 2020 Jun 25;20(2):14. doi: 10.5334/ijic.5464 (PMC7319081; doi:10.5334/ijic.5464)
Supplement: Supplementary file 2. — Topic guides. [file ijic-20-2-5464-s2.pdf]

Supplementary file 2. Topic guides

Patient/carer focus group topic guide

Timing	Questions	Prompts
12:00-12:20	**1.** Please tell me about your condition/that of the person you care for.	
	2. From your point of view, what do you think ‘coordination’ means? (*)	• If your care was coordinated (joined-up), what would it look like?<br>• Can you tell me more about that?
	3. Briefly, how are your services organised (coordinated)?<br>Specifically:<br>- Are you happy with the care coordination you experience? (*)<br>- Who organises your care? (*)	For example:<br>• How many different clinics do you have to attend?<br>• Do you see all of your doctors in the same place?<br>• Do you have a named care coordinator?
Discuss findings from scoping review		
12:20-12:30	**4.** Do you have any comments on the findings from the scoping review? (*)	• Definitions<br>• Elements<br>• Was anything (e.g. any elements) surprising to you? [1]<br>• Was anything not surprising to you?
12:30-13:25	5. Do you have any comments on xx group of features?<br>• Assessment of patient<br>• Planning<br>• Review and evaluation<br>• Support for patients<br>• Support for healthcare professionals<br>• Methods of coordination<br>• Technology<br>• Evidence-based guidelines<br>• Individual differences<br>• Wider support network<br>• Expert care<br>• Healthcare environment<br>• Administration<br>• Feedback<br>• Follow up care<br>• Coordination characteristics<br>• National policy	• Was anything surprising/not surprising?<br>• E.g. Have you used these in practice?
Comfort break and eat lunch during last part of focus group		
13:35-13:40	6. Which of the features seem most relevant/important for the coordination of care for rare conditions? (*)	• Why?<br>• Do you think the importance of these features is likely to change?<br>○ (e.g. over time/during transition?)<br>○ Different conditions / types of conditions – rare/undiagnosed

		○ Transition across services/ages
	7. Which features are important to you?	• Why?<br>• Which elements are not important?
Note. This is the topic guide for the face-to-face focus group. The virtual focus group followed similar questions but had a slightly different structure to the face-to-face focus group		

Healthcare professional focus group

Timing	Questions	Prompts
12:05-12:25 (20 minutes)	1. Please tell me about your roles/areas of work.	- Briefly, how are your services organised (coordinated) for patients and families affected by rare and undiagnosed conditions?
	2. From your point of view, if care is 'coordinated' what you do think this means?	• Can you tell me more about that?
Discuss findings from scoping review		
12:25-12:35 (10 minutes)	**3.** Do you have any comments on the findings from the scoping review?	• Definitions<br>• Elements<br>• Was anything surprising to you? [1]<br>• Was anything not surprising to you?
12:35-13:20 (40 minutes)	4. Do you have any comments on xx group of features?<br>• Assessment of patient<br>• Planning<br>• Review and evaluation<br>• Support for patients<br>• Support for healthcare professionals<br>• Methods of coordination<br>• Technology<br>• Evidence-based guidelines<br>• Individual differences<br>• Wider support network<br>• Expert care<br>• Healthcare environment<br>• Administration<br>• Feedback<br>• Follow up care<br>• Coordination characteristics<br>• National policy	- Was anything surprising/not surprising?<br>- E.g. Have you used these in practice?
13:20 – 13:30 (10 minutes)	5. Which of the features seem most relevant/important for the coordination of care for rare conditions? (*)	• Why?<br>• Do you think the importance of these features is likely to change?<br>○ (e.g. over time/during

		transition?)<br>○ Different conditions / types of conditions – rare/undiagnosed<br>○ Transition across services/ages
13:30-13:35 (5 minutes)	6. Which features are important to you?	• Why?<br>• Which elements are not important?
13:35 – 13:40 (5 minutes)	7. Are there any features of care coordination that are important to you, which are not in this table?	• If so, what are they?<br>• Why are these important?
13:40 – 13:45 (5 minutes)	8. Thinking about the scoping review findings, what do you think works well and what do you think is difficult?	• If so, which ones?<br>- Why?
13:45 – 13:55 (10 minutes)	9. Is there anything else you would like to say?	
