# Supplementary material for: Defining Coordinated Care for People with Rare Conditions: A Scoping Review
Source: Int J Integr Care. 2020 Jun 25;20(2):14. doi: 10.5334/ijic.5464 (PMC7319081; doi:10.5334/ijic.5464)
Supplement: Supplementary file 3. — References for reviews included in the scoping review. [file ijic-20-2-5464-s3.pdf]

Supplementary file 3:

References for reviews included in the scoping review

McDonald KM, Sundaram V, Bravata DM, Lewis R, Lin N, Kraft SA, et al. Closing the quality gap: a critical analysis of quality improvement strategies (Vol. 7: Care Coordination). Agency for Healthcare Research and Quality. 2007. 0051;04(07):0051-7. https://www.ncbi.nlm.nih.gov/books/NBK44015/

Yeung C, Santesso N, Zeraatkar D, Wang A, Pai M, Sholzberg M, et al. Integrated multidisciplinary care for the management of chronic conditions in adults: an overview of reviews and an example of using indirect evidence to inform clinical practice recommendations in the field of rare diseases. Haemophilia. 2016;22:41-50. https://doi.org/10.1111/hae.13010

Adli M, Bauer M, Rush AJ. Algorithms and collaborative-care systems for depression: Are they effective and why?: A systematic review. Biological psychiatry. 2006;59(11):1029-38. https://doi.org/10.1016/j.biopsych.2006.05.010

Allen J, Hutchinson AM, Brown R, Livingston PM. Quality care outcomes following transitional care interventions for older people from hospital to home: a systematic review. BMC Health Services Research. 2014;14:346. https://doi.org/10.1186/1472-6963-14-346

Archer J, Bower P, Gilbody S, Lovell K, Richards D, Gask L, Dickens C, Coventry P. Collaborative care for depression and anxiety problems. Cochrane Database of Systematic Reviews 2012, Issue 10. Art. No.: CD006525. DOI: 10.1002/14651858.CD006525.pub2.

Barr N, Vania D, Randall G, Mulvale G. Impact of information and communication technology on interprofessional collaboration for chronic disease management: a systematic review. Journal of health services research & policy. 2017;22(4):250-7. https://doi.org/10.1177/1355819617714292

Bearne LM, Byrne AM, Segrave H, White CM. Multidisciplinary team care for people with rheumatoid arthritis: a systematic review and meta-analysis. Rheumatology International. 2016;36(3):311-24. https://doi.org/10.1007/s00296-015-3380-4

Bettger JP, Alexander KP, Dolor RJ, Olson DM, Kendrick AS, Wing L, et al. Transitional care after hospitalization for acute stroke or myocardial infarction: a systematic review. Annals of internal medicine. 2012;157(6):407-16.

Betz CL, O'Kane LS, Nehring WM, Lobo ML. Systematic review: Health care transition practice service models. Nursing Outlook. 2016;64(3):229-43. https://doi.org/10.1016/j.outlook.2015.12.011

Bhawra J, Toulany A, Cohen E, Hepburn CM, Guttmann A. Primary care interventions to improve transition of youth with chronic health conditions from paediatric to adult healthcare: a systematic review. Bmj Open. 2016;6(5). http://dx.doi.org/10.1136/bmjopen-2016-011871

Binks JA, Barden WS, Burke TA, Young NL. What do we really know about the transition to adult-centered health care? A focus on cerebral palsy and spina bifida. Arch Phys Med Rehabil. 2007;88(8):1064-73. https://doi.org/10.1016/j.apmr.2007.04.018

Boland MR, Tsiachristas A, Kruis AL, Chavannes NH, Rutten-van Molken MP. The health economic impact of disease management programs for COPD: a systematic literature review and meta-analysis. BMC Pulmonary Medicine. 2013;13:40. https://doi.org/10.1186/1471-2466-13-40

Bongaerts BWC, Mussig K, Wens J, Lang C, Schwarz P, Roden M, et al. Effectiveness of chronic care models for the management of type 2 diabetes mellitus in Europe: a systematic review and meta-analysis. Bmj Open. 2017;7(3). http://dx.doi.org/10.1136/bmjopen-2016-013076

Bower P, Gilbody S, Richards D, Fletcher J, Sutton A. Collaborative care for depression in primary care: making sense of a complex intervention: systematic review and meta-regression. The British Journal of Psychiatry. 2006;189(6):484-93. https://doi.org/10.1192/bjp.bp.106.023655

Breland JY, Mignogna J, Kiefer L, Marsh L. Models for treating depression in specialty medical settings: a narrative review. Gen Hosp Psychiatry. 2015;37(4):315-22. https://doi.org/10.1016/j.genhosppsych.2015.04.010

Brooks AJ, Smith PJ, Cohen R, Collins P, Douds A, Forbes V, et al. UK guideline on transition of adolescent and young persons with chronic digestive diseases from paediatric to adult care. Gut. 2017;66(6):988-1000. http://dx.doi.org/10.1136/gutjnl-2016-313000

Burke L, Kirkham J, Arnott J, Gray V, Peak M, Beresford MW. The transition of adolescents with juvenile idiopathic arthritis or epilepsy from paediatric health-care services to adult health-care services: A scoping review of the literature and a synthesis of the evidence. Journal of Child Health Care. 2018;22(3):332-58. https://doi.org/10.1177/1367493517753330

Busetto L, Luijkx KG, Elissen AM, Vrijhoef HJ. Intervention types and outcomes of integrated care for diabetes mellitus type 2: a systematic review. Journal of Evaluation in Clinical Practice. 2016;22(3):299-310. https://doi.org/10.1111/jep.12478

Butler M, Kane RL, McAlpine D, Kathol R, Fu SS, Hagedorn H, et al. Does integrated care improve treatment for depression?: a systematic review. The Journal of ambulatory care management. 2011;34(2):113-25. doi: 10.1097/JAC.0b013e31820ef605

Cairo SB, Gasior A, Rollins MD, Rothstein DH, Amer Acad P. Challenges in Transition of Care for Patients With Anorectal Malformations: A Systematic Review and Recommendations for Comprehensive Care. Diseases of the Colon & Rectum. 2018;61(3):390-9. https://doi.org/10.1097/DCR.0000000000001033

Campbell F, Biggs K, Aldiss SK, O'Neill PM, Clowes M, McDonagh J, et al. Transition of care for adolescents from paediatric services to adult health services. Cochrane Database of Systematic Reviews. 2016;4:Cd009794. https://doi.org/10.1002/14651858.CD009794.pub2

Cape J, Whittington C, Bower P. What is the role of consultation-liaison psychiatry in the management of depression in primary care? A systematic review and meta-analysis. General Hospital Psychiatry. 2010;32(3):246-54. https://doi.org/10.1016/j.genhosppsych.2010.02.003

Chhabra PT, Rattinger GB, Dutcher SK, Hare ME, Parsons KL, Zuckerman IH. Medication reconciliation during the transition to and from long-term care settings: a systematic review. Research in social & administrative pharmacy : RSAP. 2012;8(1):60-75. https://doi.org/10.1016/j.sapharm.2010.12.002

Chu PY, Maslow GR, von Isenburg M, Chung RJ. Systematic Review of the Impact of Transition Interventions for Adolescents With Chronic Illness on Transfer From Pediatric to Adult Healthcare. Journal of Pediatric Nursing. 2015;30(5):e19-27. https://doi.org/10.1016/j.pedn.2015.05.022

Chuah FLH, Haldane VE, Cervero-Liceras F, Ong SE, Sigfrid LA, Murphy G, et al. Interventions and approaches to integrating HIV and mental health services: a systematic review. Health Policy and Planning. 2017;32(suppl_4):27-47. https://doi.org/10.1093/heapol/czw169

Coelho A, Leone C, Ribeiro V, Sa Moreira P, Dussault G. Integrated Disease Management: A Critical Review of Foreign and Portuguese Experience. Acta Medica Portuguesa. 2014;27(1):116-25.

Coffey A, Mulcahy H, Savage E, Fitzgerald S, Bradley C, Benefield L, et al. Transitional care interventions: Relevance for nursing in the community. Public Health Nursing. 2017;34(5):454-60. https://doi.org/10.1111/phn.12324

Collet J, De Vugt ME, Verhey FRJ, Schols JMGA. Efficacy of integrated interventions combining psychiatric care and nursing home care for nursing home residents: A review of the literature. International Journal of Geriatric Psychiatry. 2010;25(1):3-13. https://doi.org/10.1002/gps.2307

Coventry PA, Hudson JL, Kontopantelis E, Archer J, Richards DA, Gilbody S, et al. Characteristics of effective collaborative care for treatment of depression: a systematic review and meta-regression of 74 randomised controlled trials. PLoS One. 2014;9(9):e108114. https://doi.org/10.1371/journal.pone.0108114

Coyne B, Hallowell SC, Thompson M. Measurable Outcomes After Transfer From Pediatric to Adult Providers in Youth With Chronic Illness. The Journal of adolescent health : official publication of the Society for Adolescent Medicine. 2017;60(1):3-16. https://doi.org/10.1016/j.jadohealth.2016.07.006

Craven MA, Bland R. Better Practices in Collaborative Mental Health Care: An Analysis of the Evidence Base. Canadian Journal of Psychiatry-Revue Canadienne De Psychiatrie. 2006;51(6 Suppl 1):7S-72s.

Cronin J, Murphy A, Savage E. Can chronic disease be managed through integrated care cost-effectively? Evidence from a systematic review. Irish Journal of Medical Science. 2017;186(4):827-34. doi: 10.1007/s11845-017-1600-5)

Crowley R, Wolfe I, Lock K, McKee M. Improving the transition between paediatric and adult healthcare: a systematic review. Archives of Disease in Childhood. 2011;96(6):548-53. http://dx.doi.org/10.1136/adc.2010.202473

Cucciare MA, Coleman EA, Timko C. A conceptual model to facilitate transitions from primary care to specialty substance use disorder care: a review of the literature. Primary Health Care Research & Development. 2015;16(5):492-505. DOI: https://doi.org/10.1017/S1463423614000164

Dallimore DJ, Neukirchinger B, Noyes J. Why is transition between child and adult services a dangerous time for young people with chronic kidney disease? A mixed-method systematic review. Plos One. 2018;13(8). https://doi.org/10.1371/journal.pone.0201098

Damery S, Flanagan S, Combes G. Does integrated care reduce hospital activity for patients with chronic diseases? An umbrella review of systematic reviews. Bmj Open. 2016;6(11):e011952. http://dx.doi.org/10.1136/bmjopen-2016-011952

Davies GP, Williams AM, Larsen K, Perkins D, Roland M, Harris MF. Coordinating primary health care: an analysis of the outcomes of a systematic review. Medical Journal of Australia. 2008;188(8):S65-S8. https://doi.org/10.5694/j.1326-5377.2008.tb01748.x

de Bruin SR, Baan CA, Struijs JN. Pay-for-performance in disease management: a systematic review of the literature. BMC Health Services Research. 2011b;11:272. https://doi.org/10.1186/1472-6963-11-272

de Bruin SR, Heijink R, Lemmens LC, Struijs JN, Baan CA. Impact of disease management programs on healthcare expenditures for patients with diabetes, depression, heart failure or chronic obstructive pulmonary disease: a systematic review of the literature. Health Policy. 2011;101(2):105-21. https://doi.org/10.1016/j.healthpol.2011.03.006

de Bruin SR, Versnel N, Lemmens LC, Molema CCM, Schellevis FG, Nijpels G, et al. Comprehensive care programs for patients with multiple chronic conditions: A systematic literature review. Health Policy. 2012;107(2-3):108-45. https://doi.org/10.1016/j.healthpol.2012.06.006

Desmedt M, Vertriest S, Hellings J, Bergs J, Dessers E, Vankrunkelsven P, et al. Economic Impact of Integrated Care Models for Patients with Chronic Diseases: A Systematic Review. Value in health : the journal of the International Society for Pharmacoeconomics and Outcomes Research. 2016;19(6):892-902. https://doi.org/10.1016/j.jval.2016.05.001

Doug M, Adi Y, Williams J, Paul M, Kelly D, Petchey R, et al. Transition to adult services for children and young people with palliative care needs: a systematic review. Archives of Disease in Childhood. 2011;96(1):78-84. http://dx.doi.org/10.1136/bmjspcare.2009.163931rep

Drewes HW, Steuten LM, Lemmens LC, Baan CA, Boshuizen HC, Elissen AM, et al. The effectiveness of chronic care management for heart failure: meta-regression analyses to explain the heterogeneity in outcomes. Health services research. 2012;47(5):1926-59. https://doi.org/10.1111/j.1475-6773.2012.01396.x

Ehrlich C, Kendall E, Muenchberger H, Armstrong K. Coordinated care: what does that really mean? Health & Social Care in the Community. 2009;17(6):619-27. https://doi.org/10.1111/j.1365-2524.2009.00863.x

Ekers D, Murphy R, Archer J, Ebenezer C, Kemp D, Gilbody S. Nurse-delivered collaborative care for depression and long-term physical conditions: a systematic review and meta-analysis. Journal of affective disorders. 2013;149(1-3):14-22. https://doi.org/10.1016/j.jad.2013.02.032

Elissen AM, Steuten LM, Lemmens LC, Drewes HW, Lemmens KM, Meeuwissen JA, et al. Meta-analysis of the effectiveness of chronic care management for diabetes: investigating heterogeneity in outcomes. Journal of Evaluation in Clinical Practice. 2013;19(5):753-62. https://doi.org/10.1111/j.1365-2753.2012.01817.x

Farooq S. Collaborative care for depression: a literature review and a model for implementation in developing countries. International Health. 2013;5(1):24-8. https://doi.org/10.1093/inthealth/ihs015

Feltner C, Jones CD, Cené CW, Zheng Z-J, Sueta CA, Coker-Schwimmer EJ, et al. Transitional care interventions to prevent readmissions for persons with heart failure: a systematic review and meta-analysis. Annals of internal medicine. 2014;160(11):774-84. doi: 10.7326/M14-0083

Flanagan S, Damery S, Combes G. The effectiveness of integrated care interventions in improving patient quality of life (QoL) for patients with chronic conditions. An overview of the systematic review evidence. Health & Quality of Life Outcomes. 2017;15:1-11. https://doi.org/10.1186/s12955-017-0765-y

Franx G, Dixon L, Wensing M, Pincus H. Implementation strategies for collaborative primary care-mental health models. Current Opinion in Psychiatry. 2013;26(5):502-10. doi: 10.1097/YCO.0b013e328363a69f

Fraser MW, Lombardi BM, Wu SY, Zerden LD, Richman EL, Fraher EP. Integrated Primary Care and Social Work: A Systematic Review. Journal of the Society for Social Work and Research. 2018;9(2):175-215. https://doi.org/10.1086/697567

Fuller JD, Perkins D, Parker S, Holdsworth L, Kelly B, Roberts R, et al. Effectiveness of service linkages in primary mental health care: a narrative review part 1. BMC health services research. 2011;11(1):72. https://doi.org/10.1186/1472-6963-11-72

Gallagher C, Elliott AD, Wong CX, Rangnekar G, Middeldorp ME, Mahajan R, et al. Integrated care in atrial fibrillation: A systematic review and meta-analysis. Heart. 2017;103(24):1947-53. http://dx.doi.org/10.1136/heartjnl-2016-310952

Garralda E, Hasselaar J, Carrasco JM, Van Beek K, Siouta N, Csikos A, et al. Integrated palliative care in the Spanish context: a systematic review of the literature. BMC Palliative Care. 2016;15:1-17. https://doi.org/10.1186/s12904-016-0120-9

Gensichen J, Beyer M, Muth C, Gerlach F, Von Korff M, Ormel J. Case management to improve major depression in primary health care: a systematic review. Psychological medicine. 2006;36(1):7-14. DOI: https://doi.org/10.1017/S0033291705005568

Gilbody S, Bower P, Fletcher J, Richards D, Sutton AJ. Collaborative care for depression: a cumulative meta-analysis and review of longer-term outcomes. Archives of internal medicine. 2006;166(21):2314-21. doi:10.1001/archinte.166.21.2314

Goeman D, Renehan E, Koch S. What is the effectiveness of the support worker role for people with dementia and their carers? A systematic review. Bmc Health Services Research. 2016;16. https://doi.org/10.1186/s12913-016-1531-2

Göhler A, Januzzi JL, Worrell SS, Osterziel KJ, Gazelle GS, Dietz R, et al. A systematic meta-analysis of the efficacy and heterogeneity of disease management programs in congestive heart failure. Journal of cardiac failure. 2006;12(7):554-67. https://doi.org/10.1016/j.cardfail.2006.03.003

Haldane V, Legido-Quigley H, Chuah FLH, Sigfrid L, Murphy G, Ong SE, et al. Integrating cardiovascular diseases, hypertension, and diabetes with HIV services: a systematic review. AIDS Care. 2018;30(1):103-15. https://doi.org/10.1080/09540121.2017.1344350

Hayes SL, Mann MK, Morgan FM, Kelly MJ, Weightman AL. Collaboration between local health and local government agencies for health improvement. Cochrane database of systematic reviews (Online). 2012;10:Cd007825. https://doi.org/10.1002/14651858.CD007825.pub6

Health Quality O. Electronic tools for health information exchange: an evidence-based analysis. Ont Health Technol Assess Ser. 2013;13(11):1-76. https://www.ncbi.nlm.nih.gov/pmc/articles/PMC3814806/

Health Quality O. Continuity of care to optimize chronic disease management in the community setting: an evidence-based analysis. Ont Health Technol Assess Ser. 2013b;13(6):1-41.

Heath G, Farre A, Shaw K. Parenting a child with chronic illness as they transition into adulthood: A systematic review and thematic synthesis of parents' experiences. Patient Education & Counseling. 2017;100(1):76-92. https://doi.org/10.1016/j.pec.2016.08.011

Hoeft TJ, Fortney JC, Patel V, Unutzer J. Task-Sharing Approaches to Improve Mental Health Care in Rural and Other Low-Resource Settings: A Systematic Review. The Journal of rural health : official journal of the American Rural Health Association and the National Rural Health Care Association. 2018;34(1):48-62. https://doi.org/10.1111/jrh.12229

Homer CJ, Klatka K, Romm D, Kuhlthau K, Bloom S, Newacheck P, et al. A review of the evidence for the medical home for children with special health care needs. Pediatrics. 2008;122(4):e922-e37. doi: 10.1542/peds.2007-3762

Hopman P, de Bruin SR, Forjaz MJ, Rodriguez-Blazquez C, Tonnara G, Lemmens LC, et al. Effectiveness of comprehensive care programs for patients with multiple chronic conditions or frailty: A systematic literature review. Health Policy. 2016;120(7):818-32. https://doi.org/10.1016/j.healthpol.2016.04.002

Huang Y, Wei X, Wu T, Chen R, Guo A. Collaborative care for patients with depression and diabetes mellitus: a systematic review and meta-analysis. BMC psychiatry. 2013;13(1):260. https://doi.org/10.1186/1471-244X-13-260

Huffman JC, Adams CN, Celano CM. Collaborative Care and Related Interventions in Patients With Heart Disease: An Update and New Directions. Psychosomatics. 2018;59(1):1-18. https://doi.org/10.1016/j.psym.2017.09.003

Hussain M, Seitz D. Integrated Models of Care for Medical Inpatients With Psychiatric Disorders: A Systematic Review. Psychosomatics. 2014;55(4):315-25. https://doi.org/10.1016/j.psym.2013.08.003

Jackson GL, Powers BJ, Chatterjee R, Bettger JP, Kemper AR, Hasselblad V, et al. Patient-Centered Medical Home A Systematic Review. Annals of Internal Medicine. 2013;158(3):169-+. DOI: 10.7326/0003-4819-158-3-201302050-00579

Kamper SJ, Apeldoorn AT, Chiarotto A, Smeets R, Ostelo R, Guzman J, et al. Multidisciplinary biopsychosocial rehabilitation for chronic low back pain. Cochrane Database of Systematic Reviews. 2014(9). https://doi.org/10.1002/14651858.CD000963.pub3

Ke Y, Ng T, Chan A. Survivorship care models for breast cancer, colorectal cancer, and adolescent and young adult (AYA) cancer survivors: a systematic review. Supportive Care in Cancer. 2018;26(7):2125-41. doi: 10.1007/s00520-018-4197-y.

Kerr H, Price J, Nicholl H, O'Halloran P. Transition from children's to adult services for young adults with life-limiting conditions: A realist review of the literature. International Journal of Nursing Studies. 2017;76:1-27. https://doi.org/10.1016/j.ijnurstu.2017.06.013

Khan F, Ng L, Amatya B, Brand C, Turner-Stokes L. Multidisciplinary care for Guillain-Barre syndrome. Cochrane Database of Systematic Reviews. 2010(10). https://doi.org/10.1002/14651858.CD008505.pub2

Kooij L, Groen WG, van Harten WH. The Effectiveness of Information Technology-Supported Shared Care for Patients With Chronic Disease: A Systematic Review. Journal of Medical Internet Research. 2017;19(6):e221. doi:10.2196/jmir.7405

Kruis AL, Smidt N, Assendelft WJJ, Gussekloo J, Boland MRS, Rutten-van Molken M, et al. Integrated disease management interventions for patients with chronic obstructive pulmonary disease. Cochrane Database of Systematic Reviews. 2013(10). https://doi.org/10.1002/14651858.CD009437.pub2

Krumholz HM, Currie PM, Riegel B, Phillips CO, Peterson ED, Smith R, et al. A taxonomy for disease management: a scientific statement from the American Heart Association

Disease Management Taxonomy Writing Group. Circulation. 2006;114(13):1432-45. https://doi.org/10.1161/CIRCULATIONAHA.106.177322
Le Berre M, Maimon G, Sourial N, Gueriton M, Vedel I. Impact of Transitional Care Services for Chronically Ill Older Patients: A Systematic Evidence Review. Journal of the American Geriatrics Society. 2017;65(7):1597-608. https://doi.org/10.1111/jgs.14828
Le Roux E, Mellerio H, Guilmin-Crepon S, Gottot S, Jacquin P, Boulkedid R, et al. Methodology used in comparative studies assessing programmes of transition from paediatrics to adult care programmes: a systematic review. Bmj Open. 2017;7(1). http://dx.doi.org/10.1136/bmjopen-2016-012338
Lemmens KM, Nieboer AP, Huijsman R. A systematic review of integrated use of disease-management interventions in asthma and COPD. Respiratory medicine. 2009;103(5):670-91. https://doi.org/10.1016/j.rmed.2008.11.017
Lemmens KM, Lemmens LC, Boom JH, Drewes HW, Meeuwissen JA, Steuten LM, et al. Chronic care management for patients with COPD: a critical review of available evidence. J Eval Clin Pract. 2013;19(5):734-52. https://doi.org/10.1111/j.1365-2753.2011.01805.x
Lemmens LC, Molema CCM, Versnel N, Baan CA, de Bruin SR. Integrated care programs for patients with psychological comorbidity: A systematic review and meta-analysis. Journal of Psychosomatic Research. 2015;79(6):580-94. https://doi.org/10.1016/j.jpsychores.2015.07.013
Lewis ME, Myhra LL. Integrated Care with Indigenous Populations: A Systematic Review of the Literature. American Indian & Alaska Native Mental Health Research (Online). 2017;24(3):88-110. http://www.ucdenver.edu/academics/colleges/PublicHealth/research/centers/CAIANH/journal/Documents/Volume%2024/24(3)_Lewis_integrated_care_systematic_review_88.pdf
Lim LL, Lau ESH, Kong APS, Davies MJ, Levitt NS, Eliasson B, et al. Aspects of Multicomponent Integrated Care Promote Sustained Improvement in Surrogate Clinical Outcomes: A Systematic Review and Meta-analysis. Diabetes Care. 2018;41(6):1312-20. https://doi.org/10.2337/dc17-2010
Lion KC, Mangione-Smith R, Britto MT. Individualized Plans of Care to Improve Outcomes Among Children and Adults With Chronic Illness: A Systematic Review. Care Management Journals. 2014;15(1):11-25. http://citeseerx.ist.psu.edu/viewdoc/download?doi=10.1.1.644.9842&rep=rep1&type=pdf
Lupari M, Coates V, Adamson G, Crealey GE. 'We're just not getting it right'- how should we provide care to the older person with multi-morbid chronic conditions? Journal of Clinical Nursing. 2011;20(9-10):1225-35. https://doi.org/10.1111/j.1365-2702.2010.03620.x
MacInnes J, Williams L. A review of integrated heart failure care. Prim Health Care Res Dev. 2018:1-8.
Mackie S, Darvill A. Factors enabling implementation of integrated health and social care: a systematic review. British Journal of Community Nursing. 2016;21(2):82-7. https://doi.org/10.12968/bjcn.2016.21.2.82
Manderson B, McMurray J, Piraino E, Stolee P. Navigation roles support chronically ill older adults through healthcare transitions: a systematic review of the literature. Health

& Social Care in the Community. 2012;20(2):113-27. https://doi.org/10.1111/j.1365-2524.2011.01032.x

Martinez-Gonzalez NA, Berchtold P, Ullman K, Busato A, Egger M. Integrated care programmes for adults with chronic conditions: a meta-review. International Journal for Quality in Health Care. 2014;26(5):561-70. https://doi.org/10.1093/intqhc/mzu071

McBrien KA, Ivers N, Barnieh L, Bailey JJ, Lorenzetti DL, Nicholas D, et al. Patient navigators for people with chronic disease: A systematic review. Plos One. 2018;13(2). https://doi.org/10.1371/journal.pone.0191980

McCallum S, Mikocka-Walus A, Turnbull D, Andrews JM. Continuity of care in dual diagnosis treatment: Definitions, applications, and implications. Journal of Dual Diagnosis. 2015;11(3-4):217-32. https://doi.org/10.1080/15504263.2015.1104930

McColl MA, Shortt S, Godwin M, Smith K, Rowe K, O'Brien P, et al. Models for integrating rehabilitation and primary care: a scoping study. Arch Phys Med Rehabil. 2009;90(9):1523-31. https://doi.org/10.1016/j.apmr.2009.03.017

McIntosh D, Startsman LF, Perraud S. Mini review of integrated care and implications for advanced practice nurse role. Open Nursing Journal. 2016;10(Suppl 1: M6):78-89. doi: 10.2174/1874434601610010078

Medical Advisory Secretariat. Community-based care for the specialized management of heart failure: an evidence-based analysis. Ont Health Technol Assess Ser. 2009;9(17):1-42. https://www.ncbi.nlm.nih.gov/pmc/articles/PMC3377506/pdf/ohtas-09-42a.pdf

Miller CJ, Grogan-Kaylor A, Perron BE, Kilbourne AM, Woltmann E, Bauer MS. Collaborative chronic care models for mental health conditions: cumulative meta-analysis and metaregression to guide future research and implementation. Medical Care. 2013;51(10):922-30. doi: 10.1097/MLR.0b013e3182a3e4c4

Mitchell GK, Brown RM, Erikssen L, Tieman JJ. Multidisciplinary care planning in the primary care management of completed stroke: a systematic review. BMC Family Practice. 2008;9. https://doi.org/10.1186/1471-2296-9-44

Mitchell GK, Burridge L, Zhang J, Donald M, Scott IA, Dart J, et al. Systematic review of integrated models of health care delivered at the primary-secondary interface: how effective is it and what determines effectiveness? Aust J Prim Health. 2015;21(4):391-408. https://doi.org/10.1071/PY14172

Muntingh ADT, Van Der Feltz-Cornelis CM, Van Marwijk HWJ, Spinhoven P, Van Balkom AJLM. Collaborative care for anxiety disorders in primary care: A systematic review and meta-analysis. BMC Family Practice. 2016;17(1). https://doi.org/10.1186/s12875-016-0466-3

Ngune I, Jiwa M, McManus A, Hughes J. Do patients with long-term side effects of cancer treatment benefit from general practitioner support? A literature review. International Journal of Integrated Care. 2015;15. doi:10.5334/ijic.1987

Nicoll R, Robertson L, Gemmell E, Sharma P, Black C, Marks A. Models of care for chronic kidney disease: A systematic review. Nephrology. 2018;23(5):389-96. https://doi.org/10.1111/nep.13198

Niesink A, Trappenburg JCA, Oene G, Lammers JWJ, Verheij TJM, Schrijvers AJP. Systematic review of the effects of chronic disease management on quality-of-life in people with chronic obstructive pulmonary disease. Respiratory Medicine. 2007;101(11):2233-9. https://doi.org/10.1016/j.rmed.2007.07.017

Ouwens M, Hulscher M, Hermens R, Faber M, Marres H, Wollersheim H, et al. Implementation of integrated care for patients with cancer: a systematic review of interventions and effects. International Journal for Quality in Health Care. 2009;21(2):137-44. https://doi.org/10.1093/intqhc/mzn061

Parker S, Fuller J. Are nurses well placed as care co-ordinators in primary care and what is needed to develop their role: A rapid review? Health and Social Care in the Community. 2016;24(2):113-22. https://doi.org/10.1111/hsc.12194

Peterson K, Helfand M, Humphrey L, Christensen V, Carson S. VA Evidence-based Synthesis Program Reports. Evidence Brief: Effectiveness of Intensive Primary Care Programs. VA Evidence-based Synthesis Program Evidence Briefs. Washington (DC): Department of Veterans Affairs (US); 2011.

Peytremann-Bridevaux I, Staeger P, Bridevaux P-O, Ghali WA, Burnand B. Effectiveness of chronic obstructive pulmonary disease-management programs: systematic review and meta-analysis. The American journal of medicine. 2008;121(5):433-43. e4. https://doi.org/10.1016/j.amjmed.2008.02.009

Peytremann-Bridevaux I, Arditi C, Gex G, Bridevaux PO, Burnand B. Chronic disease management programmes for adults with asthma. Cochrane Database of Systematic Reviews. 2015(5):N.PAG-N.PAG. https://doi.org/10.1002/14651858.CD007988.pub2

Pilotto A, Cella A, Pilotto A, Daragjati J, Veronese N, Musacchio C, et al. Three Decades of Comprehensive Geriatric Assessment: Evidence Coming From Different Healthcare Settings and Specific Clinical Conditions. Journal of the American Medical Directors Association. 2017;18(2). https://doi.org/10.1016/j.jamda.2016.11.004

Pimouguet C, Le Goff M, Thiébaut R, Dartigues JF, Helmer C. Effectiveness of disease-management programs for improving diabetes care: a meta-analysis. Canadian Medical Association Journal. 2011;183(2):E115-E27. DOI: https://doi.org/10.1503/cmaj.091786

Powell Davies G, Harris M, Perkins D, Roland M, Williams A, Larsen K, et al. Coordination of care within primary health care and with other sectors: a systematic review. 2017.

Prior M, McManus M, White P, Davidson L. Measuring the "triple aim" in transition care: a systematic review. Pediatrics. 2014;134(6):e1648-61. doi: 10.1542/peds.2014-1704

Pugh JD, McCoy K, Williams AM, Bentley B, Monterosso L. Rapid evidence assessment of approaches to community neurological nursing care for people with neurological conditions post-discharge from acute care hospital. Health & Social Care in the Community. 2018;16:16. https://doi.org/10.1111/hsc.12576

Ranaghan C, Boyle K, Meehan M, Moustapha S, Fraser P, Concert C. Effectiveness of a patient navigator on patient satisfaction in adult patients in an ambulatory care setting: a systematic review. JBI Database of Systematic Reviews & Implementation Reports. 2016;14(8):172-218. doi: 10.11124/JBISRIR-2016-003049

Rochester-Eyeguokan CD, Pincus KJ, Patel RS, Reitz SJ. The Current Landscape of Transitions of Care Practice Models: A Scoping Review. Pharmacotherapy. 2016;36(1):117-33. https://doi.org/10.1002/phar.1685

Rodrigues CR, Harrington AR, Murdock N, Holmes JT, Borzadek EZ, Calabro K, et al. Effect of Pharmacy-Supported Transition-of-Care Interventions on 30-Day Readmissions: A Systematic Review and Meta-analysis. Annals of Pharmacotherapy. 2017;51(10):866-89. https://doi.org/10.1177/1060028017712725

Santomassino M, Costantini GD, McDermott M, Primiano D, Slyer JT, Singleton JK. A systematic review on the effectiveness of continuity of care and its role in patient satisfaction and decreased hospital readmissions in the adult patient receiving home care services. JBI library of systematic reviews. 2012;10(21):1214-59. doi: 10.11124/jbisrir-2012-56

Savic M, Best D, Manning V, Lubman DI. Strategies to facilitate integrated care for people with alcohol and other drug problems: a systematic review. Substance Abuse Treatment Prevention and Policy. 2017;12. https://doi.org/10.1186/s13011-017-0104-7

Schultz AT, Smaldone A. Components of Interventions That Improve Transitions to Adult Care for Adolescents With Type 1 Diabetes. Journal of Adolescent Health. 2017;60(2):133-46. https://doi.org/10.1016/j.jadohealth.2016.10.002

Sendall M, McCosker L, Crossley K, Bonner A. A structured review of chronic care model components supporting transition between healthcare service delivery types for older people with multiple chronic diseases. Health Information Management Journal. 2017;46(2):58-68. https://doi.org/10.1177/1833358316681687

Shah B, Forsythe L, Murray C. Effectiveness of Interprofessional Care Teams on Reducing Hospital Readmissions in Patients with Heart Failure: A Systematic Review. Medsurg Nursing. 2018;27(3):177-85. Retrieved from https://search.proquest.com/docview/2062944422?accountid=14511

Sigfrid L, Murphy G, Haldane V, Chuah FLH, Ong SE, Cervero-Liceras F, et al. Integrating cervical cancer with HIV healthcare services: A systematic review. Plos One. 2017;12(7). https://doi.org/10.1371/journal.pone.0181156

Siouta N, Van Beek K, van der Eerden ME, Preston N, Hasselaar JG, Hughes S, et al. Integrated palliative care in Europe: a qualitative systematic literature review of empirically-tested models in cancer and chronic disease. BMC Palliat Care. 2016;15:56. https://doi.org/10.1186/s12904-016-0130-7

Siouta N, van Beek K, Preston N, Hasselaar J, Hughes S, Payne S, et al. Towards integration of palliative care in patients with chronic heart failure and chronic obstructive pulmonary disease: a systematic literature review of European guidelines and pathways. BMC Palliative Care. 2016b;15:1-12. https://doi.org/10.1186/s12904-016-0089-4

Smith SM, Allwright S, O'Dowd T. Effectiveness of shared care across the interface between primary and specialty care in chronic disease management. Cochrane Database of Systematic Reviews. 2007(3). https://doi.org/10.1002/14651858.CD004910.pub2

Smith SM, Allwright S, O'Dowd T. Does sharing care across the primary-specialty interface improve outcomes in chronic disease? A systematic review. American Journal of Managed Care. 2008;14(4):213-24. https://www.ajmc.com/journals/issue/2008/2008-04-vol14-n4/apr08-3047p213-224

Smith SM, Soubhi H, Fortin M, Hudon C, O'Dowd T. Managing patients with multimorbidity: systematic review of interventions in primary care and community settings. Bmj-British Medical Journal. 2012;345. https://doi.org/10.1136/bmj.e5205

Smith SM, Soubhi H, Fortin M, Hudon C, O'Dowd T. Interventions for improving outcomes in patients with multimorbidity in primary care and community settings. Cochrane Database of Systematic Reviews. 2012b(4):N.PAG-N.PAG. https://core.ac.uk/download/pdf/60777199.pdf

Smith SM, Cousins G, Clyne B, Allwright S, O'Dowd T. Shared care across the interface between primary and specialty care in management of long term conditions. Cochrane Database of Systematic Reviews. 2017(2). https://doi.org/10.1002/14651858.CD004910.pub3

Somme D, Trouve H, Dramé M, Gagnon D, Couturier Y, Saint-Jean O. Analysis of case management programs for patients with dementia: a systematic review. Alzheimer's & Dementia. 2012;8(5):426-36. https://doi.org/10.1016/j.jalz.2011.06.004

Strand H, Parker D. Effects of multidisciplinary models of care for adult pre-dialysis patients with chronic kidney disease: a systematic review. International Journal of Evidence-Based Healthcare. 2012;10(1):53-9. https://doi.org/10.1111/j.1744-1609.2012.00253.x

Tam-Tham H, Cepoiu-Martin M, Ronksley PE, Maxwell CJ, Hemmelgarn BR. Dementia case management and risk of long-term care placement: a systematic review and meta-analysis. International journal of geriatric psychiatry. 2013;28(9):889-902. https://doi.org/10.1002/gps.3906

Thomas R, Huntley A, Mann M, Huws D, Paranjothy S, Elwyn G, et al. Specialist clinics for reducing emergency admissions in patients with heart failure: A systematic review and meta-analysis of randomised controlled trials. Heart. 2013;99(4):233-9. http://dx.doi.org/10.1136/heartjnl-2012-302313

Thota AB, Sipe TA, Byard GJ, Zometa CS, Hahn RA, McKnight-Eily LR, et al. Collaborative Care to Improve the Management of Depressive Disorders A Community Guide Systematic Review and Meta-Analysis. American Journal of Preventive Medicine. 2012;42(5):525-38. https://doi.org/10.1016/j.amepre.2012.01.019

Tricco AC, Antony J, Ivers NM, Ashoor HM, Khan PA, Blondal E, et al. Effectiveness of quality improvement strategies for coordination of care to reduce use of health care services: systematic review and meta-analysis. Canadian Medical Association Journal. 2014;186(15):E568-E78. DOI: https://doi.org/10.1503/cmaj.140289

Tummers JF, Schrijvers AJ, Visser-Meily JM. Economic evidence on integrated care for stroke patients; a systematic review. International Journal of Integrated Care. 2012;12. doi:10.5334/ijic.847

Turk E, Zaletel J, Ormstad SS, Micetic-Turk D, Isola A. Is a multi-disciplinary approach in the delivery of care for patients with Diabetes mellitus Type 2 cost effective? A systematic review. Healthmed. 2012;6(2):711-9. https://www.researchgate.net/profile/Fabio_Perazzo/publication/233747152_Phase_II_clinical_study_of_an_association_for_the_treatment_of_interstitial_cystitis_CystexR/links/0912f50b0b82dba025000000.pdf#page=375

Valentijn PP, Pereira FA, Ruospo M, Palmer SC, Hegbrant J, Sterner CW, et al. Person-Centered Integrated Care for Chronic Kidney Disease: A Systematic Review and Meta-Analysis of Randomized Controlled Trials. Clinical Journal of the American Society of Nephrology. 2018;13(3):375-86. DOI: https://doi.org/10.2215/CJN.09960917

van der Klauw D, Molema H, Grooten L, Vrijhoef H. Identification of mechanisms enabling integrated care for patients with chronic diseases: a literature review. International Journal of Integrated Care. 2014;14:e024. doi:10.5334/ijic.1127

van Dongen JJJ, van Bokhoven MA, Daniels R, van der Weijden T, Emonts W, Beurskens A. Developing interprofessional care plans in chronic care: a scoping review. Bmc Family Practice. 2016;17. https://doi.org/10.1186/s12875-016-0535-7

van Servellen G, Fongwa M, Mockus D'Errico E. Continuity of care and quality care outcomes for people experiencing chronic conditions: A literature review. Nursing & Health Sciences. 2006;8(3):185-95. https://doi.org/10.1111/j.1442-2018.2006.00278.x

Vanasse A, Courteau M, Ethier JF. The '6W' multidimensional model of care trajectories for patients with chronic ambulatory care sensitive conditions and hospital readmissions. Public Health. 2018;157:53-61. https://doi.org/10.1016/j.puhe.2018.01.007

Vedel I, Khanassov V. Transitional care for patients with congestive heart failure: a systematic review and meta-analysis. The Annals of Family Medicine. 2015;13(6):562-71. doi: 10.1370/afm.1844

Viggiano T, Pincus HA, Crystal S. Care transition interventions in mental health. Curr Opin Psychiatry. 2012;25(6):551-8. doi: 10.1097/YCO.0b013e328358df75

Wagner A, Brucker SY, Ueding E, Grober-Gratz D, Simoes E, Rall K, et al. Treatment management during the adolescent transition period of girls and young women with Mayer-Rokitansky-Kuster-Hauser syndrome (MRKHS): a systematic literature review. Orphanet journal of rare diseases. 2016;11(1):152. https://doi.org/10.1186/s13023-016-0536-6

Watson R, Parr J, Joyce C, May C, Le Couteur A. Models of transitional care for young people with complex health needs: a scoping review. Child: care, health and development. 2011;37(6):780-91. https://doi.org/10.1111/j.1365-2214.2011.01293.x

Watson LC, Amick HR, Gaynes BN, Brownley KA, Thaker S, Viswanathan M, et al. Practice-based interventions addressing concomitant depression and chronic medical conditions in the primary care setting: a systematic review and meta-analysis. Journal of Primary Care & Community Health. 2013;4(4):294-306. https://doi.org/10.1177/2150131913484040

Watt N, Sigfrid L, Legido-Quigley H, Hogarth S, Maimaris W, Otero-García L, et al. Health systems facilitators and barriers to the integration of HIV and chronic disease services: a systematic review. Health policy and planning. 2017;32(suppl_4):iv13-iv26. https://doi.org/10.1093/heapol/czw149

Wood E, Ohlsen S, Ricketts T. What are the barriers and facilitators to implementing Collaborative Care for depression? A systematic review. Journal of Affective Disorders. 2017;214:26-43. https://doi.org/10.1016/j.jad.2017.02.028

Xyrichis A, Lowton K. What fosters or prevents interprofessional teamworking in primary and community care? A literature review. Int J Nurs Stud. 2008;45(1):140-53. https://doi.org/10.1016/j.ijnurstu.2007.01.015

Yang F, Xiong Z-f, Yang C, Li L, Qiao G, Wang Y, et al. Continuity of care to prevent readmissions for patients with chronic obstructive pulmonary disease: a systematic review and meta-analysis. COPD: Journal of Chronic Obstructive Pulmonary Disease. 2017;14(2):251-61. https://doi.org/10.1080/15412555.2016.1256384

Yiu KC, Rohwer A, Young T. Integration of care for hypertension and diabetes: a scoping review assessing the evidence from systematic reviews and evaluating reporting. BMC Health Services Research. 2018;18(1):N.PAG-N.PAG. https://doi.org/10.1186/s12913-018-3290-8

Zhu QM, Liu J, Hu HY, Wang S. Effectiveness of nurse-led early discharge planning programmes for hospital inpatients with chronic disease or rehabilitation needs: a systematic review and meta-analysis. Journal of Clinical Nursing. 2015;24(19-20):2993-3005. https://doi.org/10.1111/jocn.12895

Zlateva I, Anderson D, Coman E, Khatri K, Tian T, Fifield J. Development and validation of the Medical Home Care Coordination Survey for assessing care coordination in the primary care setting from the patient and provider perspectives. Bmc Health Services Research. 2015;15:226. https://doi.org/10.1186/s12913-015-0893-1

Zwar N, Harris M, Griffiths R, Roland M, Dennis S, Powell Davies G, et al. A systematic review of chronic disease management. 2006.
