# Supplementary material for: Defining Coordinated Care for People with Rare Conditions: A Scoping Review
Source: Int J Integr Care. 2020 Jun 25;20(2):14. doi: 10.5334/ijic.5464 (PMC7319081; doi:10.5334/ijic.5464)
Supplement: Supplementary file 5. — Terms and definitions used. [file ijic-20-2-5464-s5.pdf]

Supplementary file 5. Terms and definitions used

Type of coordination	Number of reviews (%)	Review references	Example definition
Integrated care models / Integrated care	34 (22.1%)	Breland et al, 2015; Busetto et al, 2016; Butler et al, 2011; Chuah 2017; Coelho 2014; Collet 2010; Cronin 2017; Damery 2016; Desmedt et al 2016; Flanagan et al 2017; Gallagher 2017; Haldane 2018; Hussain 2014; Lemmens et al 2015; Lewis et al 2017; Lim et al, 2018; MacInnes et al 2018; Mackie et al, 2016; Martinez-Gonzalez 2014; McColl et al 2009; McIntosh et al 2016; Mitchell et al 2015; Ouwens et al, 2009; Savic et al 2017; Sigfrid et al 2017; Siouta et al 2016; Smith 2012; Smith 2012b; Siouta et al 2016b; Tummers et al, 2012; Valentijn et al 2018; Van der Klauw 2014; Watt et al, 2017; Yiu et al 2018	"A coherent set of methods and models on the funding, administrative, organisational, service delivery and clinical levels designed to create connectivity, alignment and collaboration within and between the cure and care sectors [Kodner & Spreeuwenberg, 2002]" [Cronin et al, 2017, p828]
Transition / care transition	18 (11.7%)	Allen et al 2014; Bhawra et al 2016; Bettger et al (2012); Chu et al, 2015; Coffey, 2017; Coyne 2017; Cucciare 2015; Chhabra et al 2012; Doug et al, 2011; Feltner et al 2014; LaBerre, 2017; Manderson et al 2012; Rochester-Eyeguokan et al 2016; Rodrigues et al, 2017; Sendall et al, 2017; Vanasse et al 2018; Viggiano et al 2012; Vedel et al, 2015	"The transition period encompasses multiple steps in a process including thoughtful planning, the actual transfer from paediatric to adult care, and adjustment to the new system afterwards" [Bhawra et al, 2016, p1]
Transition – child to adult	15 (9.7%)	Betz et al 2016, Binks et al, 2007; Brooks et al, 2017; Burke et al, 2017; Cairo et al, 2018; Campbell et al, 2016; Crowley 2011; Dallimore 2018; Heath 2017; Kerr 2017; LeRoux et al 2017; Schultz et al 2017; Prior et al 2014; Wagner 2016; Watson et al, 2011	"Health care transition has been defined as "purposeful, planned movement of adolescents and young adults with chronic physical and medical conditions from child-centered to adult-oriented health care systems that is uninterrupted, coordinated, developmentally appropriate, psychosocially sound, and comprehensive" (Blum et al, 1993, p.570)" [Betz et al, 2016, p230]
Collaborative care	15 (9.7%)	Adli et al, 2006; Archer et al 2012; Bower et al, 2006; Coventry 2014; Craven 2006; Farooq 2013; Franx 2013; Gilbody et al, 2006; Huang et al, 2013; Hayes et al 2012; Huffman 2018; Miller et al 2013; Muntingh 2016; Thota 2012; Wood et al 2017;	"Collaborative care involves providers from different specialities, disciplines, or sectors working together to offer complementary services and mutual support, to ensure that individuals receive the most appropriate service from the most appropriate provider in the most suitable location, as quickly as necessary, and with minimal obstacles."[Craven et al, 2006, p9]
Disease management programs	14 (9.1%)	Boland et al 2013; de Bruin et al 2011; de Bruin et al 2011b; Elissen et al 2013; Gohler et al, 2006; Kruis et al 2013; Krumholz 2006; Lemmens et al, 2009; Medical advisory secretariat 2009; Niesink et al, 2007; Peytremann-Bridevaux et al 2015; Peytremann-Bridevaux et al 2008; Pimouguet et al 2011; Zwar et al, 2006	"Disease management programs are multidisciplinary approaches to care for chronic disease that co-ordinate comprehensive care strategies along the disease continuum and across healthcare delivery systems. (Gonseth et al, 2004)" [Medical Advisory Secretariat, 2009, p5]
Care coordination	9 (5.8%)	Davies 2008; Ehrlich 2009; Ekers et al 2013; McDonald 2007; Mitchell et al 2008; Tricco 2014;	"Care coordination is the deliberate organization of patient care activities

Supplementary file 5. Terms and definitions used

		Parker et al 2016; Powell Davies et al, 2017; Zlateva et al 2015;	between two or more participants (including the patient) involved in a patient's care to facilitate the appropriate delivery of health care services. Organizing care involves the marshalling of personnel and other resources needed to carry out all required patient care activities, and is often managed by the exchange of information among participants responsible for different aspects of care." [McDonald et al, 2007, p5]
Case management / patient navigator	8 (5.2%)	Gensichen et al, 2006; Goeman 2016; Lupari et al, 2011; McBrien et al 2018; Pugh et al 2018; Ranaghan et al 2016; Somme et al, 2012; Tam-Tham et al, 2013	"Case management was defined as a nurse providing targeted care to individual patients which included support/clinical and social support, assessment, planning, implementation and monitoring or organising care provision to prevent and/or minimise exacerbations in the individual's chronic condition(s) (DH, 2005)" [Lupari et al, 2011, p1227]
Shared care	7 (4.6%)	Kooij 2017; Ngune et al 2015; Smith et al 2017; Smith 2007; Smith 2008; Van Dongen et al 2016; Yu et al 2018;	"Shared care is a means to improve integration and is defined as "the joint participation of GPs and hospital consultants in the planned delivery of care for patients with a chronic condition, informed by an enhanced information exchange over and above routine discharge and referral letters" [Hickman, Drummond & Grimshaw, 1994]". [Kooij et al, 2017, p2]
Continuity of care	6 (3.9%)	Health quality Ontario 2013; Health Quality Ontario 2013; McCallum et al 2015; Santomassino et al 2012; Van Servellen 2006; Yang et al, 2017;	"CoC as a one-dimensional outcome measure referring to the successful linkage of a patient from one level of care to another and CoC as an overarching construct referring to a multidimensional series of care practices during treatment" [McCallm et al, 2015, p223]
Interprofessional collaboration/ interprofessional teams	5 (3.3%)	Barr et al 2017; Fraser et al 2018; Garralda 2016; Shah et al 2018; Xyrichis et al 2008	"These benefits accrue from greater system integration and continuity of patient care by replacing professional silos with a cooperative team approach" [Barr et al, 2017, p1]
Multidisciplinary team	4 (2.6%)	Bearne et al, 2016, Pilotto et al, 2017, Strand et al, 2012; Yeung et al, 2016;	"An MDT intervention was defined as a team involving two or more health and social care professionals working in a coordinated way." [Bearne et al, 2016, p312]
Multidisciplinary	3 (2.0%)	Bongaerts et al, 2017; Khan et al, 2010; Turk et al, 2012	"Multidisciplinary care in GBS refers to delivery of co-ordinated care with

care			clearly identified goals within a specified time period, utilising at least two disciplines (medicine, physiotherapy, occupational therapy, dietetics and other allied health professions). The subcomponents of multidisciplinary care include (Stiens 1997): • an individualised patient-centred plan formulated by the patient and treating team; • goals derived and prioritised through an interdisciplinary process; • active patient participation to achieve the goals and to optimise a patient's personal potential; • assessment of outcomes specifically to determine any reductions in impairments, disability and limitation in participation." [Khan et al, 2010, p3]
Comprehensive care programs	2 (1.3%)	De Bruin et al 2012; Hopman 2016;	"Comprehensive care programs can be defined as those initiatives that proactively seek to structure and coordinate care and improve health outcomes while constraining healthcare expenditures (de Bruin et al, 2012). Many different terms are being applied to comprehensive care including integrated care, guided care, case management and shared care'. Core elements of these initiates are: a) a well-coordinated and proactive approach to health and social care needs, often including comprehensive needs assessment, b) patient-centredness by involving patients in decision-making and planning their care process, and by taking their individual needs into account, c) (simultaneous) delivery of multiple interventions, and d) involvement of professionals from multiple disciplines" [Hopman et al, 2016, p819]
Chronic care model	2 (1.3%)	Drewes et al 2012; Lemmens et al 2013;	Chronic care model – "interventions consisting of >2 components of the Chronic care model" [Lemmens et al, 2013, p735]
Plans of care	1 (0.7%)	Lion et al, 2014;	"Given the lack of a standard definition or terminology around IPCs, we used the AAP concept of a written "plan of care [that] is developed by the physician, child or youth, and family and is shared with other providers, agencies and organizations involved with the care of the patient (AAP, 2002)." [Lion et al, 2014, p12]
Models of care	1 (0.7%)	Nicoll et al 2018	"a model of care must have been capable of delivering more than one type of intervention targeted at more than one aspect of disease management." [Nicoll et al, 2018, p390]
Discharge	1 (0.7%)	Zhu et al 2015	"Nurse-led early DP programmes, which consisted of initial nurse visit within

planning			48 hours of hospital admission, predischarge assessment, structured home visits and telephone follow- ups after discharge, are led by a nurse, supported by a multidisciplinary team" [Zhu et al, 2015, p2994]
Interdisciplinary care	1 (0.7%)	Peterson et al 2011	"Multi-component, interdisciplinary intensive primary care programs". [Peterson et al, 2011, p4]
Specialist clinics	1 (0.7%)	Thomas et al 2013	"Specialist clinics were defined as units providing access to multidisciplinary teams including specialist heart failure nurses, physicians or cardiologists delivering advanced diagnostic or treatment services."[Thomas et al, 2013, p233]
Practice based interventions	1 (0.7%)	Watson et al, 2013	"We defined "practice-based" as any intervention that (a) targets the care process within a system of care and (b) aims to improve depression or both depression and chronic medical conditions. Examples of practice based interventions include: coordinated care, integrated care, collaborative care. They often involve a care manager. Each of these terms has varying, and possibly overlapping definitions and is not specifically defined for the purposes of this report. In general, we perceive them broadly to mean primary care providers and mental health providers working together to address the comprehensive needs of the patient" [Watson et al, 2013, p295]
Linkage	1 (0.7%)	Fuller et al, 2011	"A primary mental health care linkage was defined as follows: 1. The linkage is the process used to connect two or more services in the provision of clinical primary mental health care. 2. One part of the linkage must involve a primary health care practitioner such as a GP, community nurse or practice nurse. The other part of the linkage can be any health or human service entity including hospital or community based mental health specialists, private practitioners, or non-health agencies such as housing, education or welfare etc. Linkages must be two-way which excludes a single referral without feedback or continuing relationship." [Fuller et al, 2011, p2]
Medical home	1 (0.7%)	Homer et al, 2008	"These data and the experience of families led to the formulation of a model of family-centered, community-based care for CSHCN termed "the medical home" (MH) (American Academy of Pediatrics, 1992; Sia & Jacob, 1992; Sia, Tonnings, Osterhus, & Taba, 2004)." [Homer et al, 2008, p922]
Consultation	1 (0.7%)	Cape et al 2010;	"Consultation-liaison was defined as an intervention where patients were

liaison			seen once or twice by a mental health professional for assessment (consultation) and advice to the GP about management (liaison), but where no treatment was provided by the mental health professional." [Cape et al, 2010, p247]
Task sharing	1(0.7%)	Hoeft 2018;	"We conceptualize task sharing not as a referral to other providers (eg, to an urban mental health specialist without involving a local provider) but instead a sharing of care among rural providers or between rural and urban providers" [Hoeft, 2018, p49]
Patient centred medical home	1 (0.7%)	Jackson 2013	Definition of the patient-centered medical home:<br>"1. Team-based care, defined as a team-based structure in which 2 or more clinicians work together to provide care. The team may be virtual. 2. The intervention includes ≥2 of the following 4 elements: i. Enhanced access to care (e.g. advanced electronic communications, such as internet or telephone visits, open-access scheduling, group visits, 24/7 coverage). Ii. Coordinate care (care coordinated across settings, such as inpatient and outpatient, or across speciality and nonspeciality care, such as mental health, or subspecialty medicine and primary care; care management; or referral tracking). Iii. Comprehensiveness- that is, care that is accountable for addressing a large majority of personal health needs (e.g., preventive care, acute care, chronic disease care, and mental health). iv. A systems-based approach to improving quality and safety (E.g., care planning process, evidence-based medicine/clinical guidelines, point-of-care resources, electronic prescribing, test tracking, performance measurement, self-management support, accountability, and shared decision making). 3. A sustained partnership and personal relationship over time oriented toward the whole person (e.g., designating a primary point of contact who coordinates care, a personal physician, and shared decision making). 4. The intervention involves structural changes to the traditional practice, reorganizing care delivery (e.g., new personnel, new role definitions, functional linkages with community organizations and/or other health care entities, such as hospitals, specialists or other service providers, and disease registries)." [Jackson 2013, p170 figure 1]
Multidisciplinary	1(0.7%)	Kamper et al, 2014	"MBR was defined as an intervention that involves a physical component (for

Supplementary file 5. Terms and definitions used

biopsychosocial rehabilitation	example an exercise program) and at least one other element from the biopsychosocial model, that is psychological or social and occupational. The intervention program had to have been delivered by clinicians from different disciplines that is a minimum of two healthcare professionals from different professional backgrounds had to be involved in the intervention delivery. The different components of the intervention had to be offered as an integrated program involving communication between the providers responsible for the different components. We expected clinicians would include physicians, psychologists, physiotherapists, social workers, occupational therapists and exercise therapists.” [Kamper et al, 2014, p7]
