# Supplementary material for: Defining Coordinated Care for People with Rare Conditions: A Scoping Review
Source: Int J Integr Care. 2020 Jun 25;20(2):14. doi: 10.5334/ijic.5464 (PMC7319081; doi:10.5334/ijic.5464)
Supplement: Supplementary file 6. — Themes, sub-themes, components and type of components identified from reviews. [file ijic-20-2-5464-s6.pdf]

Supplementary file 6. Themes, sub-themes, components and type of components identified from reviews

Theme	Sub-theme (n=number of reviews, %)	Components (n=number of reviews, %)	Type of component		
			**What?**	**How?**	**Contextual factors**
1. Care pathway (including analysis and decision-making)	Assessment of patient and carer (n=100, 64.9%)	• Physical and mental health status (n=58, 37.7%)	X		
		• Assessment of personal factors (e.g., Challenges, knowledge, patient understanding, preferences, goals, support, barriers, risk, activities of daily living, motivation and self-management) (n=35, 22.7%)	X	*	
		• Screening (physical and mental health) (n=31, 20.1%)	X		
		• Needs (physical and mental health, neighbourhood) (n=27, 17.5%)	X	X	
		• Environment (e.g. work, home, social situation) (n=22, 14.3%)	X		
		• Medication and medication adherence (n=22, 14.3%)	X		
		• Resources (n=6, 3.9%)	X		
		• Process and outcome measurements (n=6, 3.9%)	X		
		• Discharge assessment (n=5, 3.3%)	X		
		• Readiness to engage (n=4, 2.6%)	X		
		• Readiness to transfer (n=4, 2.6%)			X
		• Self-assessment (n=3, 2.0%)	X		
		• Caregiver wellbeing, capabilities, support (n=3, 2.0%)	X		
		• Diagnostic testing (n=3, 2.0%)	X		
		• Behavioural evaluation (n=1, 0.7%)	X		
		• Eligibility (n=1, 0.7%)	X		
	Planning (n=111, 72.1%)	• Development of care plan/treatment plan, medication plan, maintenance plan, relapse prevention plan, follow up plan, discharge plan, action plan, self-management plan (n=95, 61.7%)	X	X	
		• Joint goal-setting/Goal-setting / shared decision making (n=50, 32.5%)	X	X	
		• Agreeing care with patient/carer (n=20, 13.0%)	X	X	
		• Planning who is responsible for which aspects of care (n=18, 11.7%)		X	
		• Preparation (n=10, 6.5%)		X	
		• Condition specific passport (n=5, 3.3%)	X	X	
		• Care contract between provider and patient (n=1, 0.7%)	X	X	
		• Financial planning (n=1, 0.7%)	X	X	
	Review and evaluation (n=95, 61.7%)	• Monitoring (including reviewing/progress with care plan, symptoms, progress, performance, treatment, adherence, watchful waiting and outcomes and tele monitoring) (n=79, 51.3%)	X	X	
		• Identifying and/or addressing problems/problem solving/relapse prevention (n=38, 24.7%)	X	X	
		• Medication review (n=28, 18.2%)	X		
		• Self-monitoring of behaviours/outcomes (n=24, 15.6%)	X		

			• Amending plan, care or goal to overcome difficulties (n=21, 13.6%)	X	X	
			• Evaluation (n=18, 11.7%)	X	X	
			• Clinical review/monitoring (n=11, 7.1%)	X		
		Feedback (n=48, 31.2%)	• Feedback for healthcare provider (n=28, 18.2%)	X	X	
			• Feedback (non-specific) (includes benchmarking) (n=18, 11.7%)	X		
			• Feedback to patients (n=9, 5.8%)	X		
			• Feedback from patients/carers about care (n=9, 5.8%)	X	X	
			• Feedback from healthcare providers (n=4, 2.6%)	X	X	
			• Management of patient complaints (n=1, 0.7%)	X		
			• Biofeedback (n=1, 0.7%)	X		
		Follow up (n=91, 59.1%)	• Follow up (e.g. conducted over telephone/face to face/web/mailing) (n=81, 52.6%)	X		
			• Post discharge follow up (n=31, 20.1%)	X		
			• Follow up arranged with patient (n=15, 9.7%)	X		
			• Structured/systematic follow up/used register to follow up (n=13, 8.4%)	X		
			• Pharmacotherapy /medication follow up (n=2, 1.3%)	X		
			• No formal follow up (n=1, 0.7%)	X		
			• Follow up of test results (n=1, 0.7%)	X		
		Administration (n=62, 40.3%)	• Reminders (n=41, 26.6%)	X		
			• Documentation / record keeping / reports (n=29, 18.8%)	X	X	
			• Appointment scheduling (n=24, 15.6%)	X	X	
			• Administrative support (n=7, 4.6%)	X	X	
			• Client consent (n=1, 0.7%)			X
			• Technological difficulties / safeguarding support (n=1, 0.7%)	X		
2.	Approaches	Methods of coordination (n=149, 96.8%)	• Team approach (e.g. multi/interdisciplinary) (n=118, 76.6%)		X	
			• Coordination/collaboration/case management/disease management/integrated approach (n=109, 70.8%)		X	
			• Responsibility of coordination by one person (e.g. coordinator/care managers/care led by one provider or set of providers) (n=109, 70.8%)		X	
			• Communication between providers, other providers and/or patients (n=88, 57.1%)		X	
			• Using and sharing documentation /info (e.g. patient records/notes/medical summary) (n=72, 46.8%)		X	
			• Single visit approach (including colocation, specific clinics and medical homes) (n=62, 40.3%)		X	
			• Referral systems (n=57, 37.0%)		X	
			• Meetings to bring team together to discuss coordination (n=48, 31.2%)		X	
			• Transition (n=41, 26.6%)		X	
			• Task sharing/delegation/shared care (n=29, 18.8%)		X	
			• Patient/carer coordinating own treatment / patient carer involvement / patients as partners in care /patient held		X	

			records (n=26, 16.9%)		
			• Continuity of care (n=23, 14.9%)		X
			• Joint clinics/consultation (n=23, 14.9%)		X
			• Alternating appointments/visits (n=5, 3.3%)		X
			• Professional communities or practices (n=4, 2.6%)		X
			• None/lack of coordination (n=3, 2.0%)		X
		Expert care (n=97, 63.0%)	• Specialist care/specialist teams/expert knowledge/condition specific expertise/specialist referrals/expert review of guidelines (n=89, 57.8%)	X	
			• Specialist clinics /condition-specific clinics (n=35, 22.7%)		X
			• Patient and family expertise (n=2, 1.3%)	X	
			• Lack of expertise (n=1, 0.7%)	X	
		Technology (n=93, 60.4%)	• Telecare (including Telehealth/home based monitoring system /use of monitoring equipment or technological aids, telemedicine/electronic prescriptions/tele psychiatry) (patients/carers) (n=37, 24.0%)	X	X
			• Algorithms/decision support aids for HCPs (n=35, 22.7%)		X
			• Communication systems/ teleconferencing (HCPs) (n=35, 22.7%)		X
			• Electronic medical records/personal health records/continuity of care records (n=34, 22.1%)		X
			• Clinical information systems (HCPs) (e.g. patient tracking etc.) (n=32, 20.8%)		X
			• Reminders/recall and alert system (HCPs) (n=31, 20.1%)	X	X
			• Centralised database of patients/registry (HCPs) (n=28, 18.2%)		X
			• Automated performance monitoring (HCPs) / feedback systems (n=24, 15.6%)	X	
			• Support/education using technology e.g. via online platforms (patients/carers) /e-consultations (n=24, 15.6%)	X	
			• Non-specific IT (n=19, 12.3%)	X	
			• Use of online platform (HCPs) (e.g. online learning/general IT/websites/IT platforms/shared IT platforms /computerised lab records) (n=18, 11.7%)	X	X
			• Electronic appointment reminders (patients/carers) (n=11, 7.1%)	X	X
			• Automated summaries and reports and other materials (HCPs) (n=10, 6.5%)		X
			• Delivery of questionnaires (HCPs, patients, carers) (n=6, 3.9%)	X	
			• Technological support (n=4, 2.6%)	X	
			• Use of other digital tools e.g. digital camera (n=1, 0.7%)	X	
3.	Support	Support provided to patients and carers (n=144, 93.5%)	• Education and information for patients / skills training (understanding condition and management, transition; honest, open and age appropriate; available services; support transition; communication skills) (n=113, 73.4%)	X	X
			• General support for patients (including social support, practical support, non-specific support, general support for transition, and support from different modalities, e.g. face to face / letter / phone /email/newsletters) (n=105, 68.2%)	X	X
			• Self-management support (n=84, 54.6%)	X	X

			• Psychological support (e.g. counselling/CBT/MI, emotional support, behavioural therapy) (n=77, 50%)	X		
			• Medical treatment (including support for medication, medical treatment, surgery) (n=73, 47.4%)	X		
			• Home visits (n=62, 40.3%)	X	X	
			• Physical health support (e.g. Diet, exercise, smoking cessation, health promotion) (n=54, 35.1%)	X		
			• Involvement of social workers/other community personnel (including volunteers) (n=54, 35.1%)	X	X	
			• Signposting/linking to community resources/ community based referrals/community based care (n=51, 33.1%)	X	X	
			• Support for carers, including education and information (e.g. (how they can support their child, information about coordination, sectors) (n=40, 26.0%)	X	X	
			• Information about /support for accessing services/resources or using healthcare aspects e.g. records (n=25, 16.2%)	X	X	
			• Support from pharmacist (n=25, 16.2%)	X	X	
			• Peer mentoring/involvement of peers/mentor programs/peer support/peer educators (n=24, 15.6%)	X		
			• Opportunities to become familiar with coordinated approach (E.g. visits, tours, lunch, joint visits) (n=15, 9.7%)		X	
			• Family support / involvement (n=11, 7.1%)	X	X	
			• Palliative care support (n=7, 4.6%)	X		
			• Support with other aspects of care e.g. Social welfare benefits/legal services/finances, housing (n=6, 3.9%)	X		
			• Consultations out of clinic (n=3, %)	X	X	
			• Social networking (n=2, 1.3%)	X		
			• Genetic counselling (n=1, 0.7%)	X		
		Support provided to health care professionals (n=85, 55.2%)	• Education (including summer camps) (n=50, 32.5%)	X	X	
			• Training (n=48, 31.2%)	X	X	
			• Supervision/support (n=47, 30.5%)		X	
			• Support tools (n=10, 6.5%)	X	X	
			• Non-specific interventions (n=6, 3.9%)	X	X	
			• Administrative / technical support (n=5, 3.3%)	X	X	
			• Behaviour plans (n=1, 0.7%)		x	
			• Capacity building (n=1, 0.7%)	X	X	
4.	Features	Individual differences (n=69, 44.8%)	• Individualised care/care plans / patient centred care (n=51, 33.1%)			X
			• Taking into account culture, health, demographic factors (e.g. age or gender), readiness to transition, goals and expectations, independence (n=35, 22.7%)			X
			• Flexible (Healthcare/timing) (n=12, 7.8%)			X
		Evidence-based practice (n=100, 64.9%)	• Guideline based treatment (n=57, 37.0%)			X
			• Evidence-based standardised treatment protocols (n=54, 35.1%)			X
			• Standardised /structured care (including use of manual, checklists/competency frameworks/criteria) (n=30, 19.5%)			X
			• Evidence-based pathways/evidence based care/evidence-based screening (n=28, 18.2%)			X
			• Treatment algorithms (n=22, 14.3%)			X

			• Evidence-based tools (n=8, 5.2%)			X
			• Best practice/previous treatments/recommendations (n=8, 5.2%)			X
			• Policy/policy template (n=7, 4.6%)			X
			• Unstructured/non guideline based care (n=1, 0.7%)			X
			• Treatment targets (n=1, 0.7%)			X
		Other (n=31, 20.1%)	• Qualified staff (n=11, 7.1%)			X
			• Length of intervention (n=9, 5.8%)			X
			• Amount of contact (n=3, 2.0%)			X
			• Interested/willing providers (n=3, 2.0%)			X
			• Type of coordination (n=2, 1.3%)			X
			• Characteristics of staff (n=2, 1.3%)			X
5.	Wider environment	Healthcare environment (n=87, 56.5%)	• Supportive environment for coordination (e.g. distance from treatment facilities/access to care) (n=48, 31.2%)			X
			• Resources (e.g. expertise, staffing, resources) (n=33, 21.4%)			X
			• Quality improvement/evaluation of services (n=31, 20.1%)	X		
			• Financial incentives (including providers, institution and patients) (n=27, 17.5%)		X	
			• Organisation of healthcare system (including service planning…) (n=26, 16.9%)		X	
			• Structural changes (n=25, 16.2%)		X	
			• Organisational support (including support from organisation and agreements/discussions between organisations and professional networks) (n=25, 16.2%)			X
			• Outreach (n=17, 11.0%)	X		
			• Governance (n=9, 5.8%)	X	X	
			• Identification of barriers to care/assessment of need (n=8, 5.2%)	X		
			• Use of existing services (n=2, 1.3%)	X		
			• Lack of supportive environment (n=1, 0.7%)	X		
		Wider environment (n=15, 9.7%)	• National policy changes (n=9, 5.8%)			X
			• Campaigns (n=5, 3.3%)			X
			• Funding collaborations/changes to funding (n=2, 1.3%)			X
			• Geographical coverages and rostering (n=2, 1.3%)			X

Note: Those components that fall into both what and how have been identified with an 'x' in both columns. This table provides a summary of the components that make up coordinated care.
* One study (McDonald et al, 2007) highlights assessment can be used to identify coordination challenges
