# Supplementary material for: Defining Coordinated Care for People with Rare Conditions: A Scoping Review
Source: Int J Integr Care. 2020 Jun 25;20(2):14. doi: 10.5334/ijic.5464 (PMC7319081; doi:10.5334/ijic.5464)
Supplement: Supplementary file 7. — Example quotes from the stakeholder consultations for ‘what’, ‘how’ and ‘facilitating components’. [file ijic-20-2-5464-s7.pdf]

Supplementary file 7. Example quotes from the stakeholder consultations for 'what', 'how' and 'facilitating components'

Type of components	Sub-theme	Example quote(s)
Components that need to be coordinated during a patients' care pathway ('what')	Support for patients/carers and healthcare professionals	*"¶689: Well, the first thing that [Name 2] was diagnosed when he was seven and the day that I got in touch with the [Organisation 4] which was a group of parents doing, you know, that was the most important thing, that was the most important day of my life and when I say my life I mean that, not just my son's life." (FG-PC2)*<br><br>*"¶487: Not only for us but for the professionals as well because if they are uneducated or unaware of certain things, they need the support just as much as we do to sort of be able to treat us and allow us to get the care that we need." (FG-PC1)*
	Elements of care that need to be coordinated	*"¶195: I'm thinking of review and evaluation with the therapist and on in-community. So, for people with rare conditions, there'll be a period of intervention, then all will go quiet, everything's being managed well, and then some other problem will come up, but the way therapists work is that there'll be a period of intervention, measured outcome, closed case, no contact kept until a crisis down the road and they come back, and when I was in that position, I always wanted to be able to keep that person under review […] because you were then managing a situation before it became a crisis." (FG-HCP)*<br><br>*"Yeah definitely for me, definitely for my son diagnosis has changed everything, I feel completely empowered to look after him because all I need to say are the words [Condition 1] and then people go away they've got Google they can find… Prior to that I felt completely alone with it and like I said before, I felt like a fraud even my own family didn't really until he got diagnosed really believed that there could possibly be something so randomly wrong. And so I think diagnosis is key and we only [cuts off] the genome 100,000 scheme that's why he got diagnosed and, you know, we're eternally grateful for that really." (FG-PC1)*
Components that tell us how to coordinate care ('how')	Coordination through taking responsibility	*"¶207: We do need leadership. If this was in a theatre, you know, if you were having an operation the surgeon's in charge, it's the same with this, it's no different really, somebody has got to be in charge, I've actually had to tell doctors you are the main man, until you make a decision the other three aren't going to do anything so I think this thing about coordination is very, very important."* (FG-PC2)<br><br>*"¶229: I think having a consultant who takes the lead has been the best thing for me, that's been the most helpful because I ring his secretary for everything and he knows that it's his responsibility and he took responsibility but just for himself, he didn't do it because he's being paid to do it he just recognises that we were really sinking." (FG-PC1)*<br><br>*"¶575: Well, very similar I mean when it comes to coordination I think it's about, for me anyway, I'd like to be in partnership with somebody rather than me doing it all but also because these sorts of illnesses, disorders, whatever, there is very little I can control about them and this might be the only thing I can have some control over but, yeah." (FG-PC2)*<br><br>*"¶631: And I do wonder with rare conditions, whether there ought to be an advocate all the way through. So, somebody in maternity, somebody in special care, somebody on the paediatric ward, somebody in the community nurse team, the health visitor, that there's just one person, so that that person can link to that person that can link to that person, almost." (FG-HCP)*

	Coordination through specialist centres	*"¶458: That's absolutely got to be gold standard for any of those kinds of conditions. To really have, if you're talking about your evaluation, your reassessment, your planning and implementation, and getting that real, you know, assurance about that follow-up, you know, to do that annually, do an MDT, I mean…" (FG-HCP)*<br><br>*"¶215: […] He actually does attend a rare disease clinic and there is some attempt at coord- so he's actually seen three doctors on the same day." (FG-PC2)*
	Coordination through technology	*"¶304: P4: And, again, if there is one system, it would be good if everybody does it, you know.[…]*<br>*¶307: Interviewer: All of the different NHS trusts?*<br>*¶308: P4: Yeah. Everyone's doing their own things at the moment.*<br>*¶309: P3: I think the key is that systems talk to each other because it's so time-consuming and arduous to get the information to the right people in an efficient way. It takes hours on just getting it right, and things are going to get missed, and it isn't good enough, and I think technology could be something that could be much easier to join up, you would imagine." (FG-HCP)*
	Coordination through communication	*"¶614: [Name 2] has a health passport that it takes into hospital with him but it's very basic, it's about his condition and it's about certain things, he doesn't like this, he doesn't like that because he's no communication but I think everybody really, everybody should have one of those even if you've only got a basic condition that when you're going into hospital you've got something that you can say, yes, that's me." (FG-PC2)*<br><br>*"¶114: you know, you should have access and all those people should speak to each other because it's an interconnected condition and they don't, in fact it's quite hard to find a single person who knows." (FG-PC1)*
	Coordination through support	*"¶410: […] my son has a rare condition that no-one knows anything about and so therefore there's no support group, there's no charity group, there's nothing for my daughter, you know, who watches my son in hospital week after week after week, I'm away for stretches at a time, there's nothing in place and I think that is part of co-ordinated care because that's care for my family." (FG-PC1)*<br><br>*"¶720: so the defining moment really and truly is having somebody that knew about the condition, you know, okay we've got a diagnosis but the help that we got and the ability to say we need or we want come from that, and this is why I would suggest to anybody join a support group, go to the association if there is one and if there's not an association or a support group make one which, you know? That's my contribution." (FG-PC2)*<br><br>*¶266: Yeah, and if the school's been set up, I always find, then the parents truly get some respite whilst the child's there because they don't have to worry about them the whole time if they know the care is being, continuing to be coordinated there on a safe basis, so I think that's a really important(?) support." (FG-HCP)*<br><br>*"¶292: I know, from a family point of view, we do, kind of, family integrated care on our neonatal unit where we get the parents, you know, presenting their babies on ward rounds and being involved with patients' decisions so that it becomes… if they've got that*

		experience while they're in hospital, you know, they get quite confident." (FG-HCP)
		"¶656: I always felt that transition skills to develop were more important because that person was going on to advocate for themselves and manage their own health condition, and would need to be alert and know when to go to an orthopaedic consultant, when to go to a community therapist" (FG-HCP)
	Other: Team approach	*"I think it's quite accurate on the fourth point as a team approach because I think that is it's not a team approach it's very much in depending as long as the doctor that's seeing you maybe that day gets his stuff done he's not concerned if it impacts or anything else from any of the other doctors, I think there's not a team approach in that respect whereas I think if they had more of a mentality around a team approach things might work better. And again that just goes back to anything in life, if you have one goal and you all have a common goal then you're going to have a better chance of hitting it if you're all going in the same direction than if you were going right, left, north, south, east, west, so that kind of stood out to me." (FG-PC2)*
	Other: Continuity of care	*"¶304: I think what could be useful is if you have a family history of a rare or chronic condition if you had one specialist who deals with all members of the same family because if you are clones then you are very likely to respond to the same treatment, that has happened in my family and that's very useful because you have one specialist who knows the lot of you and he then knows that if a treatment suits one of you it should suit all of you and in my case for that particular illness it has been very useful and we've all responded well to the treatment." (FG-PC1)*
	Other: Planning	*"¶100: Now we've always asked for a proper care plan and I'm sure every… I mean you were saying earlier on you've been there done that […] If you look at the rare disease strategy a care plan is probably the most common thing that patients ask for and there's a lot of reasons for that, one you can monitor health over the year, you know what you're doing and if you've got more than one issue like you've got central nervous issues or you've got liver issues or you've got movement issues it's all co-ordinated and the place you can remember and it restores compliance but the important thing it actually monitors the evolution of the rare disease and you can monitor performance. Now why there is such a resistance to a proper care plan I do not know because I've never ever been able to get one." (FG-PC2)*
		"¶1140: Yes, the only thing I'd say hasn't changed is a care plan in place [cuts off], I've still not received a care plan for him and they're still… we won't take him to [Place 3] hospital anymore, we just will not go it's too dangerous... […]…because there's nothing on their system and that has to change, it's ridiculous, it has to change that." (FG-PC1)
Components that facilitate coordination ('facilitating')	Evidence-based practice	*"¶167: […] it's, having those clear pathways… […] …and having something that people can, you know, work towards which is really clear.[…] Even though it's very rare, it's, like, 'Okay, this is the process now,' and that's really important. […] And I think that makes the systems work a lot better if there is something like that in place. I think when it's wishy-washy or it's not clear, or there is no clear, kind of, guidance or pathways, and because some of the situations are quite, you know, specialised, I think it's difficult, it's very difficult to manage." (FG-HCP)*

	Individualised treatment	*"¶282:Everybody is put into the same category, I mean me and P2 both here have [Condition 3] but I bet our stories are also very different and I find that I'm put in a box because I've got [Condition 3] I'm just seen to some doctors as being a little bit bendy and actually there's a hell of a lot more going on than just that and I find everybody is either put in that tick box scenario and we're not getting the treatment and care that we deserve and need." (FG-PC1)*<br><br>*"¶556: Every single person in this phone call every single person with a rare condition their experience(?) is different, so they may want to take these identified points, routes, identified points and they may want to either put them in a chain of command, how they want it to happen, they may want to put it as if someone's running a project on it in a project management style thing or in a hierarchy of importance with the very minimum that we need and the pinnacle being the optimum that we want or in another form of pyramid where it says the top of the hierarchy is the absolute base that we need and going down to what we want, there are various ways of actually using what are really good groups and identified elements but in a different manner."*
	Access to care	*"¶691: And like you said before, when you realise that you have to push for things you just do it and then you start doing it naturally with everything and at the beginning you got emotional about it but after a bit you don't get emotional and you just go through the motions and you go I know how it works now, you just keep going, I think remove the emotion from it because I found at the start I was picking all different things, you realise it's a game really of semantics and stuff like that" (FG-PC2)*
	Access to healthcare environment	*"¶703: The other thing that I was wondering from a parent point of view is how few specialists there are and they're all old, my age, so we don't seem to say, right, we've got this… and how medical professions develop how do you attract new talent in to actually take over from the older clinicians who are leaving? And that is a concern because we've only got four or five in the UK who even you would use to diagnosis and many rare diseases as I say." (FG-PC2)*

Note. Components included in the 'multi-purpose' category from the scoping review are discussed within both the 'what' analysis and the 'how' analysis in the consultation section. This is so that sufficient detail can be given but also so that their role for each purpose is clear.
